# A hybrid breast cancer/mesenchymal stem cell population enhances chemoresistance and metastasis

**DOI:** 10.1172/jci.insight.164216

**Published:** 2023-09-22

**Authors:** Giuseppina Augimeri, Maria E. Gonzalez, Alessandro Paolì, Ahmad Eido, Yehyun Choi, Boris Burman, Sabra Djomehri, Santhosh Kumar Karthikeyan, Sooryanarayana Varambally, Johanna M. Buschhaus, Yu-Chih Chen, Loredana Mauro, Daniela Bonofiglio, Alexey I. Nesvizhskii, Gary D. Luker, Sebastiano Andò, Euisik Yoon, Celina G. Kleer

**Affiliations:** 1 Department of Pathology, University of Michigan Medical School, Ann Arbor, Michigan, USA.; 2 Department of Pharmacy, Health and Nutritional Sciences, University of Calabria, Rende, Italy.; 3 Rogel Cancer Center and; 4 Department of Electrical Engineering and Computer Science and Department of Biomedical Engineering, University of Michigan, Ann Arbor, Michigan, USA.; 5 Department of Pathology, University of Alabama at Birmingham, Birmingham, Alabama, USA.; 6 Center for Molecular Imaging, Department of Radiology, University of Michigan, Ann Arbor, Michigan, USA.; 7 UPMC Hillman Cancer Center, Department of Computational and Systems Biology, Department of Bioengineering, University of Pittsburgh, Pittsburgh, Pennsylvania, USA.; 8 Department of Computational Medicine and Bioinformatics, University of Michigan, Ann Arbor, Michigan, USA.

**Keywords:** Cell Biology, Oncology, Breast cancer

## Abstract

Patients with triple-negative breast cancer remain at risk for metastatic disease despite treatment. The acquisition of chemoresistance is a major cause of tumor relapse and death, but the mechanisms are far from understood. We have demonstrated that breast cancer cells (BCCs) can engulf mesenchymal stem/stromal cells (MSCs), leading to enhanced dissemination. Here, we show that clinical samples of primary invasive carcinoma and chemoresistant breast cancer metastasis contain a unique hybrid cancer cell population coexpressing pancytokeratin and the MSC marker fibroblast activation protein-α. We show that hybrid cells form in primary tumors and that they promote breast cancer metastasis and chemoresistance. Using single-cell microfluidics and in vivo models, we found that there are polyploid senescent cells within the hybrid cell population that contribute to metastatic dissemination. Our data reveal that Wnt Family Member 5A (WNT5A) plays a crucial role in supporting the chemoresistance properties of hybrid cells. Furthermore, we identified that WNT5A mediates hybrid cell formation through a phagocytosis-like mechanism that requires BCC-derived IL-6 and MSC-derived C-C Motif Chemokine Ligand 2. These findings reveal hybrid cell formation as a mechanism of chemoresistance and suggest that interrupting this mechanism may be a strategy in overcoming breast cancer drug resistance.

## Graphical abstract

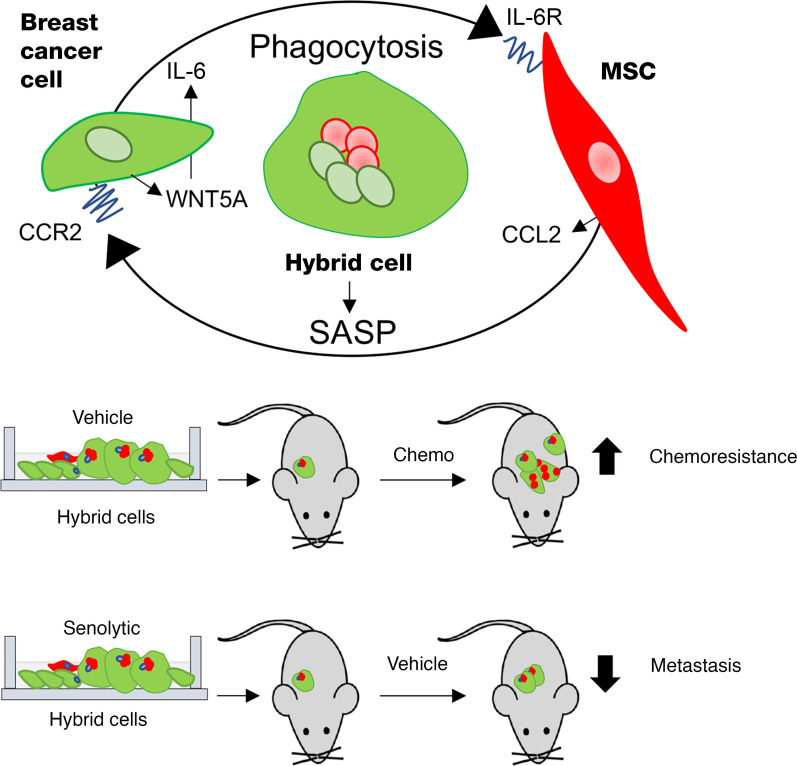

## Introduction

Despite advances in breast cancer treatment, breast cancer is still a leading cause of mortality in women worldwide (1). One of the major limitations of breast cancer therapy is the establishment of resistance to chemotherapy, which leads to patients’ relapse and death (2). In particular, triple-negative breast cancer (TNBC) frequently recurs after an initial response to chemotherapy (3). Elucidating the mechanisms underlying breast cancer resistance to treatment is needed to improve survival.

The crosstalk between breast cancer cells (BCCs) and the components of the tumor microenvironment has emerged as a crucial modulator of the effectiveness of drug therapy (4). BCCs communicate with stromal cells through paracrine loops and through cell-to-cell and cell-to-matrix interactions, creating a permissive tumor microenvironment that facilitates cancer cell escape to drug treatment (5). Mesenchymal stem/stromal cells (MSCs) are self-renewing pluripotent cells that are recruited from the bone marrow and the adipose tissue to the primary tumor site by soluble molecules released by BCCs (6). In response to the local signals produced by BCCs, MSCs release factors that support resistance to therapy and breast cancer progression (7–9). Additionally, MSCs can contribute to the development of breast cancer chemoresistance through physical interaction with BCCs (10). Our lab and others have demonstrated that MSCs can be engulfed by BCCs, supporting breast tumorigenesis (11, 12). We have isolated and characterized patient-derived MSCs from human breast cancer metastasis, and we have shown that MSC engulfment by BCCs induces heritable transcriptional changes resulting in a hybrid cell population with enhanced metastatic ability (11). However, the phenotypic features of hybrid cells and their role in breast cancer progression are not well understood.

Here, using unbiased RNA-Seq studies, we demonstrate that MSCs derived from patients’ metastasis express fibroblast activation protein-α (FAP) and identify and quantify hybrid cells coexpressing cytokeratin (CK) and FAP in clinical samples of primary tumors and distant metastasis. We provide direct evidence that MSC engulfment by BCCs generates hybrid cells in orthotopic tumors and lung metastases using 2-photon microscopy. We found that the hybrid cell population is enriched in senescent cells and displays a chemoresistant phenotype. Our studies identify that MSC engulfment utilizes a phagocytosis-like mechanism that requires Wnt Family Member 5A–mediated (WNT5A-mediated) IL-6 and CCL2 secretion. These data provide the foundation to regulate hybrid cell formation as a potential strategy to overcome breast cancer chemoresistance.

## Results

### Primary breast carcinomas and distant metastasis contain a population of hybrid cancer cells expressing mesenchymal stem/stromal cell markers.

**Figure 1:**
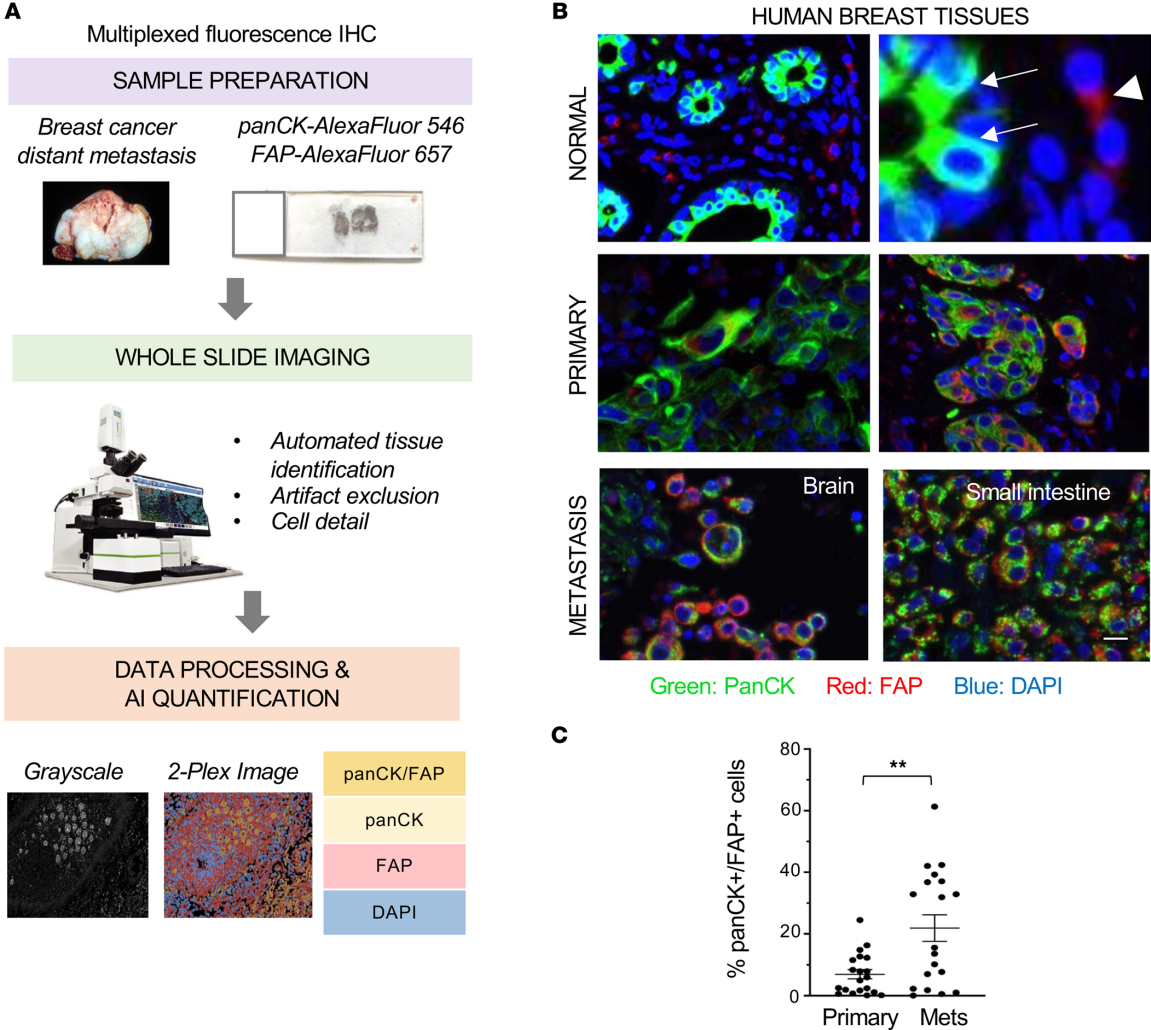
Analysis of clinical samples using quantitative multiplex fluorescence immunostaining. (**A**) Workflow for multiplex image analysis. We included clinical samples from primary breast cancers (*n* = 20) and breast cancer distant metastasis (*n* = 18) using pancytokeratin (pan-CK), the mesenchymal cell marker fibroblast activation protein-α (FAP), and DAPI. For data processing and quantification, we used an automated artificial intelligence (AI) system. (**B**) Representative images of human breast samples showing the presence of hybrid cancer cells coexpressing pan-CK and FAP. In normal breast tissue, pan-CK is expressed by epithelial cells in the acini (arrows) and FAP is expressed in the stromal cells (arrowhead). In contrast, primary invasive carcinomas and metastasis contain a population of cancer cells with a hybrid pan-CK^+^/FAP^+^ phenotype. Scale bar: 30 µm. (**C**) Quantification of the percentage of pan-CK^+^/FAP^+^ cells as well as total cells analyzed per sample. Data are expressed as individual values with mean ± SEM analyzed with 2-tailed unpaired Student’s *t* test. **P < 0.005.

We have shown that BCCs can engulf MSCs, generating a hybrid cancer cell population with phenotypic epithelial-to-mesenchymal transition (EMT) and expression of EMT proteins (e.g., Zinc Finger E-Box Binding Homeobox 1 [ZEB1] and smooth muscle actin [SMA]) as well as greater metastatic ability than nonengulfing BCCs (11). To identify specific markers that allow detection of BCCs, MSCs, and hybrid BCCs that have engulfed MSCs in human samples, we performed RNA-Seq of MDA-MB-231 BCCs and MSCs isolated from patient metastasis previously characterized in our lab (13). We identified FAP as one of the most significantly upregulated proteins in MSCs with undetectable expression in MDA-MB-231 cells, and this was further validated by Western blot (WB) (Supplemental Figure 1, A and B; supplemental material available online with this article; https://doi.org/10.1172/jci.insight.164216DS1). Next, we sought to determine the presence of BCCs expressing a hybrid phenotype in 38 clinical tissue samples, including 20 primary invasive carcinomas with adjacent normal breast and 18 distant breast cancer metastasis that have failed chemotherapy. We employed quantitative multiplexed fluorescence IHC with pan-CK, FAP antibodies, and an artificial intelligence (AI) pipeline that allows quantification of specific cell populations (Figure 1A). In normal breast lobules, we observed a clear separation between pan-CK^+^ breast epithelial cells and FAP^+^ stromal cells. In contrast, primary invasive carcinomas and distant metastases contain a unique population of cells that coexpress pan-CK and FAP, indicating a hybrid phenotype (Figure 1B, Supplemental Figure 1C, and Supplemental Tables 1 and 2). Quantification of the percentage of pan-CK^+^/FAP^+^ cells in whole tissue sections showed that distant metastases have a significantly higher percentage of hybrid cells compared with primary tumors (Figure 1C).

To detect the formation of hybrid cells in vivo, we injected GFP-labeled MDA-MB-231 cells (GFP-231) and DsRed-labeled MSCs (DsRed-MSCs) in the mammary fat pads of NOD/SCID mice. Using in vivo 2-photon fluorescence microscopy of orthotopic tumors and ex vivo imaging of lungs, we identified GFP^+^/DsRed^+^ hybrid cells in orthotopic tumors and in spontaneous distant lung metastasis (Supplemental Figure 2A). In addition, to investigate the metastatic potential of the hybrid cells, we sorted by flow cytometry the GFP^+^/DsRed^+^ cells from the hybrid cell–enriched coculture of GFP-231 with DsRed-MSCs and intracardially injected the GFP^+^/DsRed^+^ in NOD/SCID mice. We observed that hybrid cells can form metastasis that grow over time using bioluminescence imaging (Supplemental Figure 2B). Together, these data provide direct evidence that hybrid cells form in situ in live mammary tumors, have the ability to disseminate, and can be detected in tissue samples of primary breast cancer and metastasis using a combination of pan-CK and FAP markers.

### Generation and characterization of hybrid cells in cocultures of BCCs and mesenchymal stem cells.

**Figure 2:**
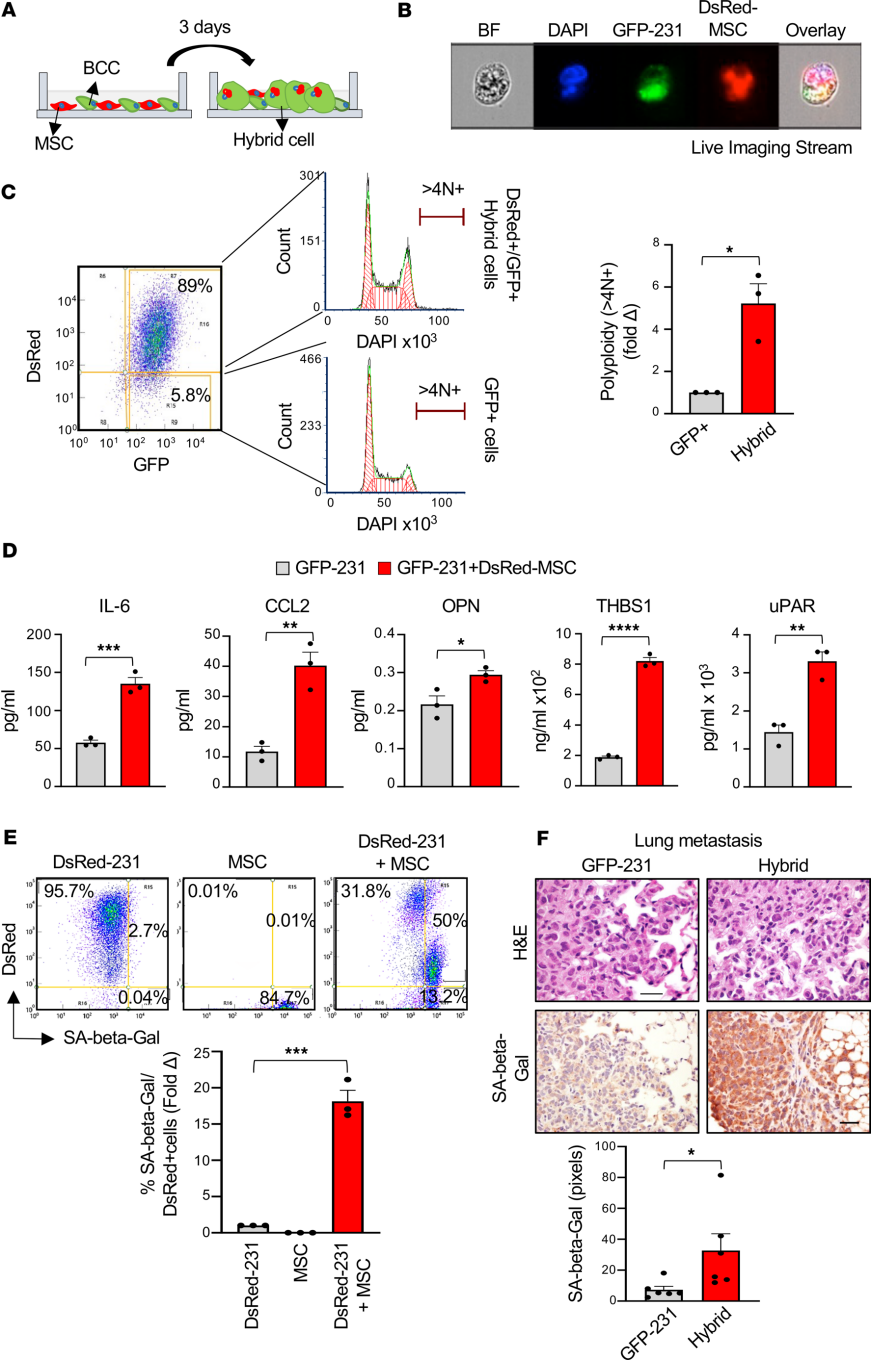
MSC engulfment by breast cancer cells (BCCs) generates a hybrid polyploid population characterized by a senescent phenotype. (**A**) Schematic representation of BCCs cultured with MSCs to obtain hybrid-enriched population. (**B**) Representative flow cytometry live imaging stream pictures showing a Ki67^lo^ hybrid cell. GFP-labeled MDA-MB-231 (GFP-231) BCCs were cultured with DsRed-labeled MSCs (DsRed-MSCs) for 72 hours and stained with Ki67 antibody. Ki67^lo^ cells were sorted and fixed, and nuclei were stained with DAPI. An overlay of all fluorescence channels shows a multinucleated GFP^+^/DsRed^+^ hybrid cell. Cell phase image (bright-field [BF]) is included to display cell morphology. (**C**) Cell-cycle analysis by flow cytometry in the coculture of GFP-231 BCCs with DsRed-MSCs after 72 hours showing the emergence of a polyploid population in GFP^+^/DsRed^+^ (hybrid) compared with GFP^+^ cells. Bar graph shows the quantification of the flow cytometry analyses. (**D**) ELISA analyses of IL-6, CCL2, OPN, THBS1, and uPAR in supernatants of GFP-231 BCCs and MSCs diluted 1:1 (GFP-231) or in coculture (GFP-231 + DsRed-MSC). (**E**) Senescence-associated β-Gal (SA–β-Gal) activity analyzed by flow cytometry using DsRed-labeled MDA-MB-231 cells (DsRed-231) and MSC (unlabeled) in single cultures and cocultures for 1 week**.** Bar graph shows the percent of SA–β-Gal/DsRed^+^ cells. (**F**) Lung metastases from mice injected intracardially with GFP-231 or GFP^+^/FAP^+^ hybrid cells sorted by FACS, stained with H&E and SA–β-Gal by IHC (*n* = 3 per group). Bar graph shows the quantification of the SA–β-Gal in 5 different fields per condition (*n* = 4 per group) using ImageJ. Scale bars: 20 µm. For all panels, data are shown as individual values with mean ± SEM. In **C**, **D**, and **F**, 2-tailed unpaired Student’s *t* test was employed; in **E**, 1-way ANOVA with Tukey’s multiple comparison test was employed. *P < 0.05; **P < 0.005; ***P < 0.0005; ****P < 0.0001.

We cocultured a panel of GFP-labeled BCCs, including MDA-MB-231 (GFP-231), MDA-MB-436 (GFP-436), and patient-derived T4 BCCs (14) with DsRed-MSCs for 72 hours (Figure 2A). In addition, we generated GFP-CCN6–KO BCCs from MMTV-Cre;Ccn6^fl/fl^ mammary breast carcinomas, which recapitulate the histopathology and genomic features of human spindle metaplastic TNBC (15, 16) and cultured GFP-CCN6–KO cells with DsRed-MSCs for 7 days.

**Figure 3:**
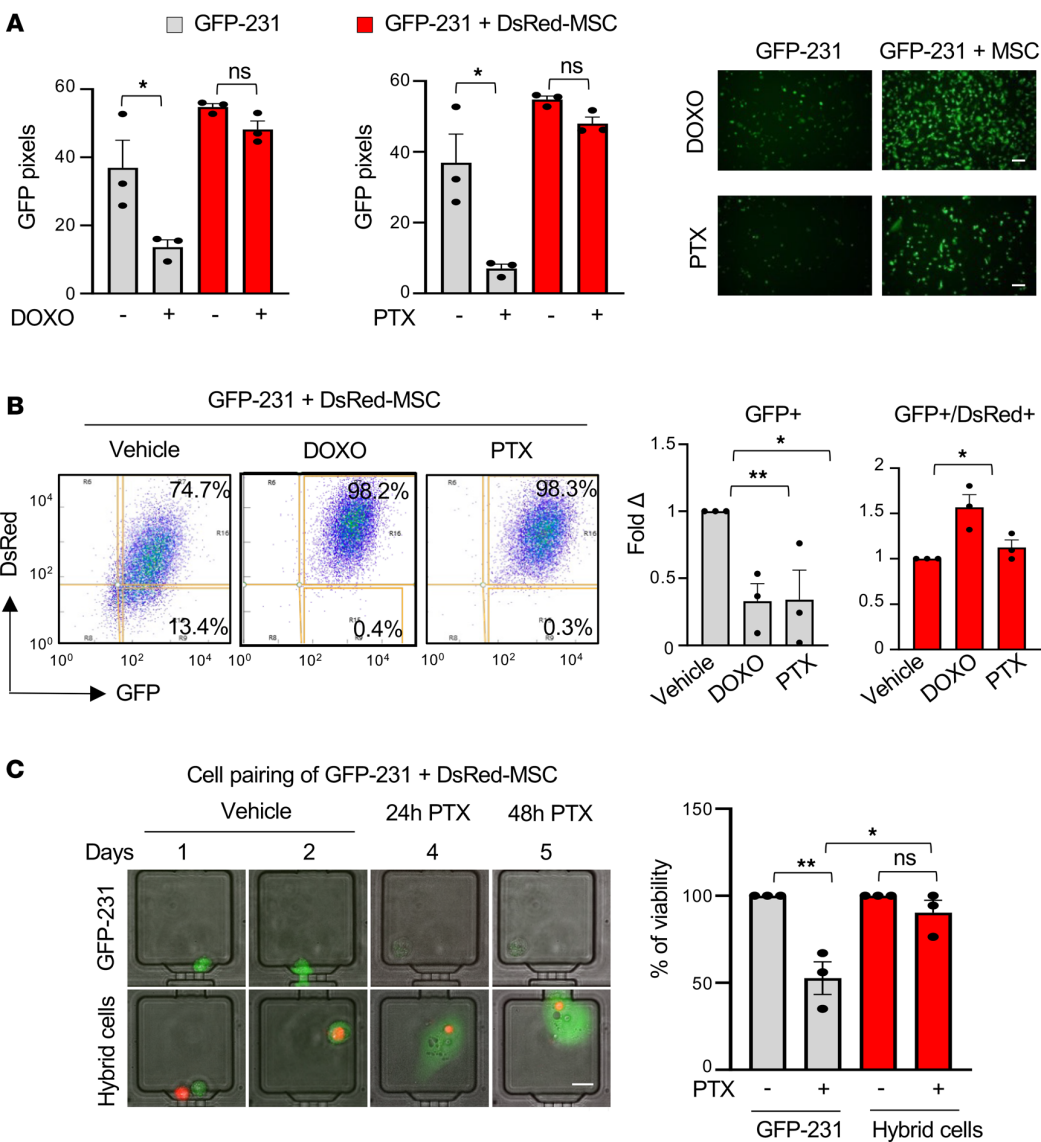
Hybrid cells exhibit chemoresistance properties. (**A**) GFP signal of GFP-231 cells alone or in coculture with DsRed-MSCs, treated with doxorubicin (DOXO) 1 μM, paclitaxel (PTX) 10 μM, or vehicle for 24 hours. GFP was quantified in 3 different fields per condition using ImageJ. Representative pictures of GFP signal upon drug treatment are shown. Scale bar: 200 mm. (**B**) Effect of chemotherapy on the specific GFP^+^ and GFP^+^/DsRed^+^ populations of cocultures of GFP-231 with DsRed-MSCs treated with DOXO 1 μM, PTX 10 μM, or vehicle. Bar graphs show the quantification of flow cytometry analyses expressed in fold changes of each population of cells with respect to vehicle-treated samples. (**C**) A single GFP-231 alone or with a DsRed-MSC was loaded in the microfluidic device. After 72 hours, cells were treated with PTX 10 μM for 48 hours (days 4 and 5). Representative images of hybrid cells and GFP-231 breast cancer cells are shown. Scale bar: 20 μm. Note that PTX treatment induced reduced cell viability only in GFP-231 cells. Bar graph shows the percent of cell viability ± SEM of hybrid cells and GFP-231 after 48 hours of PTX treatment (*n* = 10, in biological triplicates). For all panels, data are expressed as individual values with mean ± SEM analyzed. In **A** and **C**, 2-way ANOVA with Tukey’s multiple comparison test was applied; in **B**, 1-way ANOVA with Tukey’s multiple comparison test was applied. *P < 0.05; **P < 0.005.

Flow cytometry live imaging stream single-cell analysis showed the presence of hybrid cells in cocultures of GFP-231 and DsRed-MSCs (Figure 2B) and 3-dimensional renderings of confocal *Z* stacks (Supplemental Video 1). In concordance with our in vivo observations (Supplemental Figure 2A), hybrid cells characterized by GFP and DsRed signals were found in the cocultures of GFP-436, GFP-T4, and GFP-CCN6–KO BCCs with DsRed-MSCs analyzed by immunofluorescence microscopy (Supplemental Figure 2C).

Coculture of GFP-231 with MSCs resulted in 89% GFP^+^/DsRed^+^ hybrid cells (Figure 2C), and coculture of GFP-CCN6–KO cells with DsRed-MSC cells for 7 days led to 95% hybrid cells (Supplemental Figure 2D), representing well-suited experimental cell models to investigate the role of hybrid cells in breast cancer progression.

### Hybrid cells exhibit a senescent phenotype.

In order to characterize the hybrid cell population that we identified in vivo and in vitro, we first investigated the presence of DNA copy number changes in hybrid cells analyzing their DNA content by flow cytometry. We found that, in the coculture of GFP-231 with DsRed-MSCs, the GFP^+^/DsRed^+^ hybrid cells are polyploid with increased DNA content (greater than tetraploid DNA content, >4N^+^) compared with GFP^+^ cells (Figure 2C). Since polyploidization is often a feature of senescence, we hypothesized that hybrid cells may display a senescent phenotype (17–19). We used cytokine arrays and ELISA to compare the secretome profiles of hybrid-enriched cocultures, BCCs, and MSCs. Compared with the supernatant of BCCs and MSCs mixed in the ratio 1:1, hybrid-enriched cocultures exhibit increased levels of several senescence-associated secretory phenotype (SASP) factors, including IL-6, C-C motif chemokine ligand 2 (CCL2), osteopontin (OPN), thrombospondin 1 (THBS1), and urokinase-type plasminogen activator receptor (uPAR) (Figure 2D and Supplemental Figure 3A) (12, 20–22). Similar results were observed using GFP-436 and GFP-T4 patient-derived BCCs (Supplemental Figure 3, B and C). To investigate whether the direct contact between BCCs and MSCs is required for secretion of SASP factors, DsRed-MSCs were cultured physically divided from GFP-231 using a transwell system followed by analysis of the supernatants by cytokine array (Supplemental Figure 3D). We found a reduced secretion of the SASP factors in the supernatants of GFP-231 and DsRed-MSCs cultured in the transwell system compared with the direct contact coculture, demonstrating that the direct contact between BCCs and MSCs is necessary for secretion of SASP.

We next measured the senescence-associated β-galactosidase (SA–β-Gal) activity, a well-characterized marker of cellular senescence (23), in cocultures of DsRed-labeled MDA-MB-231 (DsRed-231) with unlabeled MSCs. Flow cytometry analysis (Figure 2E) and SA–β-Gal staining (Supplemental Figure 4, A and B) revealed an increased percentage of DsRed^+^ cells expressing SA–β-Gal in the hybrid cell–enriched coculture compared with single culture. Moreover, we observed higher SA–β-Gal staining in the metastases from mice injected with flow-sorted hybrid cells compared with GFP-231 labeled with luciferase (Luc–GFP-231) (Figure 2F). Together, these data indicate that hybrid cells are enriched in polyploid cells with a senescent phenotype.

### Hybrid cells drive breast cancer metastatic progression and chemoresistance.

The main reason for chemotherapy failure in TNBC is the emergence of chemoresistance, which ultimately leads to patient death. Thus, we investigated whether hybrid cells might contribute to breast cancer chemoresistance. Toward this, we treated hybrid cell–enriched cocultures or single cultures of GFP-BCCs with doxorubicin (DOXO) or paclitaxel (PTX), 2 first-line chemotherapeutic agents used in patients with breast cancer (24, 25). Treatment with DOXO or PTX significantly reduced the GFP signal in GFP-231, GFP-436, and CCN6-KO BCCs in single culture. However, neither drug reduced the GFP signal in the hybrid-enriched cocultures (Figure 3A and Supplemental Figure 5, A and B). To investigate in greater detail the effect of chemotherapy specifically on hybrid cells, we treated the cocultures with DOXO, PTX, or vehicle and sorted the GFP^+^ and GFP^+^/DsRed^+^ hybrid cells by flow cytometry. As shown in Figure 3B, DOXO or PTX treatment effectively reduced the percentage of GFP^+^ 231 cells compared with vehicle-treated samples. However, neither drug reduced the percentage of GFP^+^/DsRed^+^ cells, and DOXO increased the percentage of hybrid cells. We next investigated the impact of chemotherapy on each single hybrid cell using our microfluidic single-cell paring device, which allowed us to follow the formation of hybrid cells and the fate of each cell over time (11). For this, we loaded GFP-231 alone or with DsRed-MSC (1:1) in each microfluidic chamber and after 72 hours of incubation, we treated the cells with PTX for 48 hours. We observed that PTX treatment significantly reduced cell viability of GFP^+^ 231 cells without affecting cell viability of hybrid cells. Moreover, hybrid cells had higher cell viability compared with single cells upon PTX treatment. These data demonstrate that hybrid cells show chemoresistant properties at a single-cell resolution level (Figure 3C).

**Figure 4:**
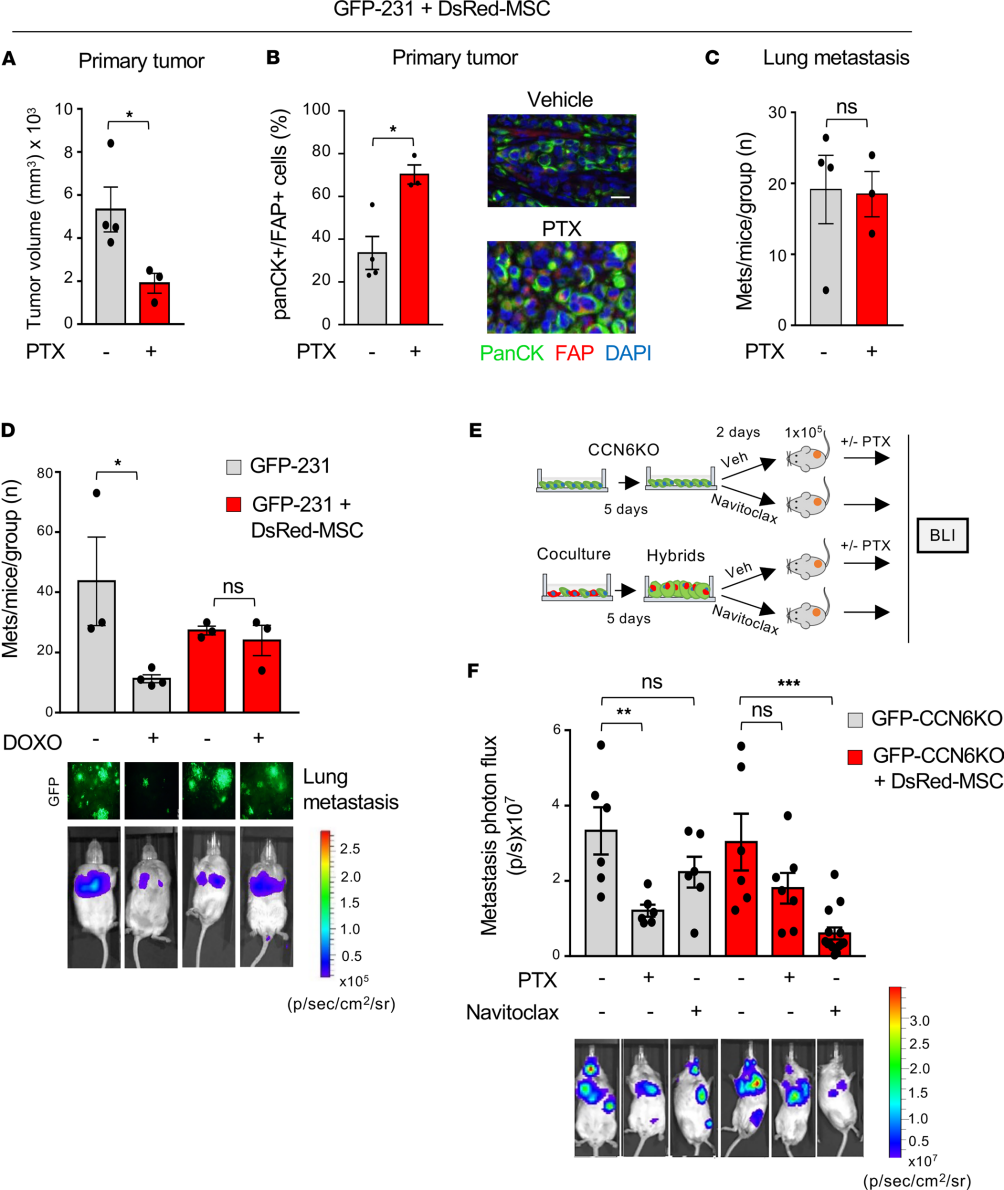
In vivo, hybrid cells drive resistance to chemotherapy and senescent hybrid cells specifically enhance metastasis. (**A**) Quantification of primary tumor volume of cocultures injected in the mammary fat pads of NOD/SCID mice treated with vehicle (–) or with PTX 10 mg/kg every 3 days i.p. for 20 days (*n* = 3–4 per group). (**B**) Primary mammary tumors in **A** were subjected to quantitative fluorescence multiplex immunostaining for pan-CK and FAP to quantify the percent of pan-CK^+^/FAP^+^ hybrid cells. Shown are representative images of vehicle- or PTX-treated tumors. Scale bar: 50 µm. (**C**) Number of spontaneous lung metastasis per mice per group of mice in **A** assessed by histopathology. (**D**) Luc–GFP-231 (1.0 × 10^5^) alone or cocultured with DsRed-MSCs for 72 hours (1.5 × 10^5^ cells) were injected intracardially in NOD/SCID mice. At day 20, mice were treated with DOXO (4 mg/kg every 3 days) or vehicle for 1 week (*n* = 3-4 per group). Shown are representative images of GFP^+^ lung metastases and bioluminescence images of distant metastases at necropsy in mice treated with DOXO or vehicle. Quantification of the number of metastases in mice using GFP pixels. Scale bar: 20 µm. (**E**) Schematic illustrating that Luc–GFP-CCN6–KO BCCs (1.0 × 10^5^) alone or with DsRed-MSC were cultured for 5 days and treated with Navitoclax (5 μM for 48 hours). Cells (1 × 10^5^) were intracardially injected in FVB mice. After 2 days control mice were treated with vehicle or PTX 10 mg/kg for 10 days and followed using bioluminescence imaging (BLI). (**F**) Representative bioluminescence images of metastases at indicated conditions (*n* = 6–14 per group). For all panels, data are presented as individual values with mean ± SEM. In **A**–**C**, the 2-tailed unpaired Student’s *t* test was employed; in **D**, 1-way ANOVA with post hoc Tukey HSD/Tukey-Kramer was used; in **F**, 2-way ANOVA with Tukey’s multiple comparison test was used. *P < 0.05; **P < 0.005; ***P < 0.0005.

Based on these results, we set out to test the ability of hybrid cells to progress during chemotherapy in the presence of primary mammary tumors, modeling the clinical setting of neoadjuvant chemotherapy. Hybrid-enriched cocultures of GFP-231 with DsRed-MSCs containing 89% hybrid cells were orthotopically inoculated into the mammary fat pads of NOD/SCID mice. When tumors became palpable, mice received PTX every 3 days. As shown in Figure 4A, PTX reduced the size of primary tumors compared with vehicle, whereas it increased the percentage of pan-CK^+^/FAP^+^ compared with vehicle-treated controls (Figure 4B), analyzed using multiplex immunofluorescence staining. Notably, in mice bearing hybrid enriched tumors, PTX had no effect on the number of spontaneous pulmonary metastases compared with controls (Figure 4C).

To directly test the ability of hybrid cells to acquire chemoresistance in vivo, Luc–GFP-231 in single culture and in cocultures with DsRed-MSCs were injected intracardially in NOD/SCID mice. We began treatment with DOXO 20 days after intracardiac injection and euthanized mice at day 27, when they developed clinical signs of metastasis. Quantification of the number of distant metastases by GFP immunofluorescence of fresh tissues at necropsy (Figure 4D) and by histopathological studies (not shown) demonstrated that, as expected, DOXO reduced the number of distant metastases in mice injected with Luc–GFP-231 cells compared with vehicle. In contrast, DOXO treatment had no significant effect on the number of metastases in mice injected with hybrid-enriched coculture compared with vehicle (Figure 4D).

Cell senescence has been linked to drug resistance and metastasis (26–29). Thus, we next investigated the chemoresistant properties of hybrid cells and the relevance of the senescent cells within the hybrid population to metastasis. For these experiments, we employed CCN6-KO BCCs in syngeneic FVB immune competent mice. We cultured CCN6-KO BCCs with DsRed-MSCs for 5 days, which resulted in 95% hybrid GFP^+^/DsRed^+^ cells (Supplemental Figure 2D), and treated the cells with Navitoclax, a senolytic drug that selectively eliminates nonproliferative cells (30). Navitoclax significantly reduced the GFP signal of hybrid cells compared with control but had no significant effect in the GFP signal of CCN6-KO cells in single culture (Supplemental Figure 5C). Next, we intracardially injected GFP-CCN6–KO cells labeled with Firefly-luciferase (Luc–GFP-CCN6–KO) and hybrid cells pretreated with Navitoclax or vehicle. Additionally, we treated the control mice with PTX or vehicle for 10 days (Figure 4, E and F). Validating the data with DOXO treatment, PTX significantly reduced distant metastases of Luc–GFP-CCN6–KO but had no significant effect on the distant metastasis of hybrid cells compared with the vehicle control group. Notably, pretreatment of hybrid cells, but not BCC single cultures, with Navitoclax significantly reduced metastatic burden compared with controls (Figure 4F). Collectively, these data show that hybrid cells enhance breast cancer metastatic progression and chemoresistance and that the senescent cells within the hybrid population are important for metastasis.

### WNT5A downregulation rescues MSC engulfment and hybrid cell chemoresistance.

**Figure 5:**
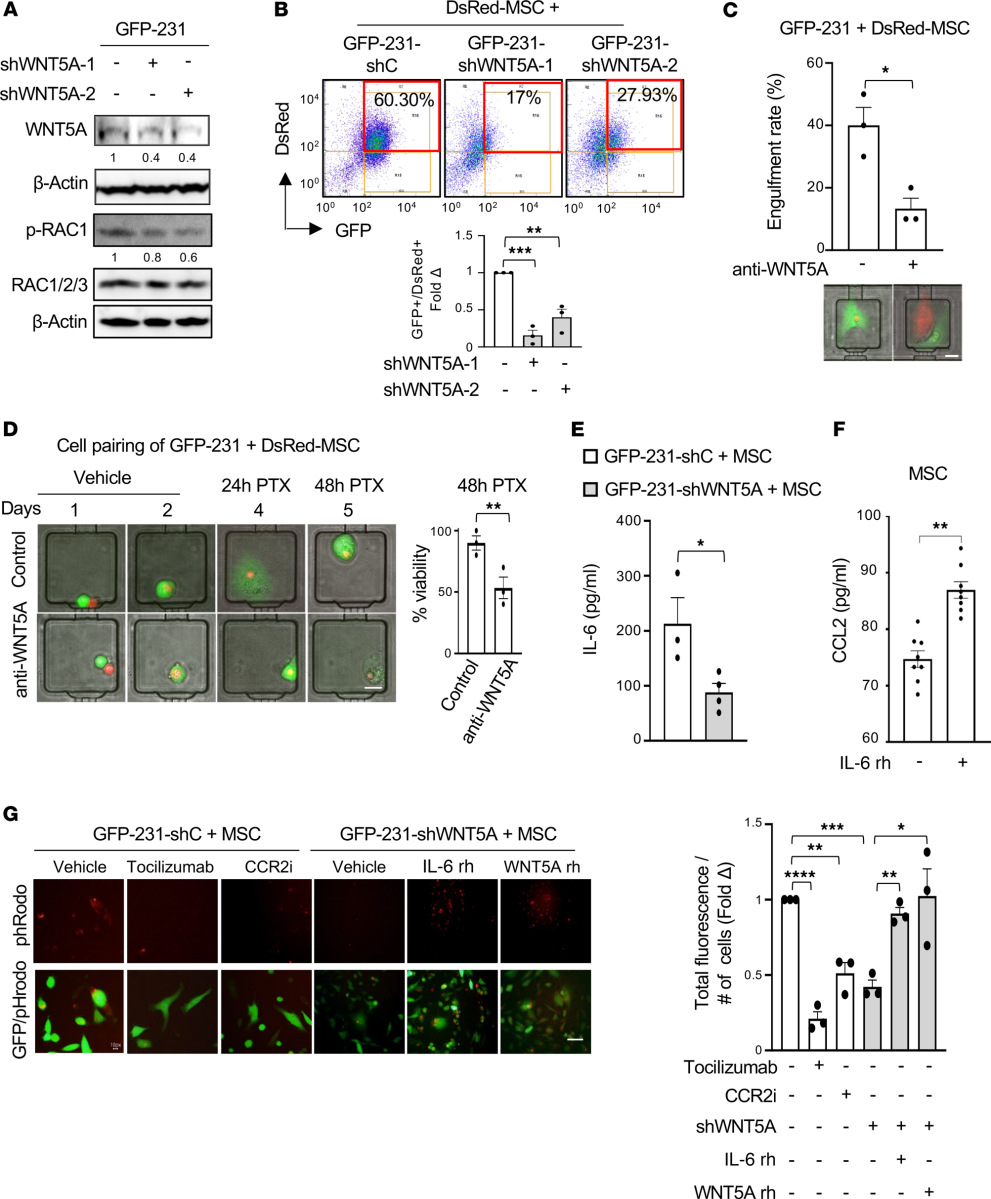
WNT5A, IL-6, and CCL2 mediate hybrid cell formation through a phagocytosis-like mechanism. (**A**) Immunoblots of GFP-231 transduced with lentivirus control (-shC) or with 2 independent shWNT5A (1 and 2). β-Actin was used as loading control. Numbers below the blots represent the average fold change over GFP-231–shC. See complete unedited blots in the supplemental material. (**B**) Representative flow cytometry of indicated cells show that WNT5A shRNA reduces the percent of GFP^+^/DsRed^+^ hybrid cells. (**C**) A single GFP-231 and a single DsRed-MSC were loaded in the microfluidic cell-pairing device and treated with vehicle or anti-WNT5A over time. Bar graph shows percent of engulfment ± SEM (*n* = 10, in biological triplicate). Representative images illustrating that anti-WNT5A inhibited hybrid cell formation. Scale bar: 20 µm. (**D**) GFP-231 and MSC loaded in the microchip and, after 3 days, were treated with PTX 1 μM for 48 hours (*n* = 10 cells/condition, in biological triplicate). Scale bar: 20 µm. (**E**) ELISA for IL-6 protein secretion in supernatants of cocultures of DsRed-MSCs and GFP-231–shC or –shWNT5A after 72 hours. (**F**) ELISA for CCL2 protein secretion in the supernatants of DsRed-MSCs treated with vehicle (–) or with IL-6 recombinant protein (rh), 30 ng/mL. (**G**) Representative pHrodo and merge fluorescence images of GFP-231–shC treated with vehicle, tocilizumab 30 μg/mL, or CCR2 inhibitor (CCR2i) 40 μM, and GFP-231-shWNT5A treated with vehicle, IL-6 rh 30 ng/mL, and WNT5A rh 1 μg/mL before incubation with pHrodo-labeled MSCs for 48 hours. Scale bar: 50 µm. Bars quantify total red fluorescence normalized to cell number. pHrodo signal was quantified in 3 fields/condition using ImageJ. For all panels, data are expressed as individual values with mean ± SEM. In **B**, 1-way ANOVA with Turkey’s multiple comparison test was employed; in **C**–**F**, 2-tailed unpaired Student’s t test; and in **G**, 2-way ANOVA with Tukey’s multiple comparison test was employed. *P < 0.05, **P < 0.005, ***P < 0.0005; ****P < 0.0001.

We have reported that MSC engulfment induces persistent gene expression changes in MDA-MB-231 cells with significant upregulation of WNT5A by hybrid cancer cells compared with nonengulfing counterparts (11). To directly investigate the role of WNT5A in hybrid cell formation, we knocked down the endogenous expression of WNT5A (short hairpin WNT5A [shWNT5A]) in GFP-231 BCCs using lentiviral delivered shRNA (Figure 5A). We also established shWNT5A in GFP-T4 patient-derived BCCs (Supplemental Figure 6A). shWNT5A effectively reduced WNT5A protein levels in both cell lines (Figure 5A and Supplemental Figure 6A) and reduced phosphorylation of WNT5A downstream targets RAC (RAC 1/2/3), Protein kinase C-α (PKC-α) and phospholipase C 1-γ-1 (PLC-1-γ-1) (31) compared with GFP-231 cells transduced with scrambled controls (shC) (Figure 5A and Supplemental Figure 6B). WNT5A shRNA downregulation or WNT5A blocking antibody significantly reduced the percentage of hybrid cells, shown with flow cytometry (Figure 5B) and immunofluorescence staining (Supplemental Figure 6, C and D), and reduced the rate of MSC engulfment measured using a microfluidics cell pairing device (Figure 5C). Demonstrating the relevance to chemoresistance, WNT5A blocking antibody reduced the viability of hybrid cells treated with PTX in the microfluidic device compared with vehicle (Figure 5D). Altogether, these data show that the downregulation of WNT5A reduces MSC engulfment and generation of hybrid cells. These results also demonstrate that WNT5A knockdown leads to phenotypic changes in the hybrid cells, reducing their chemoresistant properties.

### Hybrid cells are generated through a phagocytosis-like mechanism mediated by a WNT5A–IL-6–CCL2 cytokine network.

Studies have shown that WNT5A regulates IL-6 expression in melanoma and modulates the expression of SASP factors in tendon stem cells, and that IL-6 exhibits a reciprocal regulation with CCL2 in non–small cell lung cancer (32, 33). These data, together with our results that hybrid cell–enriched cocultures secrete SASP factors (Figure 2D), suggest the hypothesis that WNT5A may maintain the generation of hybrid cells through a cytokine network involving SASP proteins. To further examine the detailed mechanisms involved in the generation of hybrid cells, we first investigated the cell type responsible for specific SASP protein secretion. Using cytokine arrays and ELISA of the conditioned media of GFP-BCCs and DsRed-MSCs, we found that BCCs are the main source of IL-6, OPN, THBS1, and uPAR and that MSCs secrete CCL2 (Supplemental Figure 7, A–C). WNT5A downregulation in GFP-231 significantly reduced secretion of IL-6 in the conditioned media of hybrid enriched populations (Figure 5E), and IL-6 recombinant protein was sufficient to increase CCL2 secretion by MSCs (Figure 5F). Collectively, these data reveal a cytokine network between BCC and MSC in which WNT5A released by BCCs stimulates IL-6 secretion that, in turn, increases the release of CCL2 by MSCs.

Next, we investigated whether WNT5A might mediate hybrid cell formation through a phagocytosis-like mechanism. GFP-231–shC and GFP-231–shWNT5A cells were cultured with MSCs labeled with a pHrodo dye that becomes fluorescent when MSCs enter in the acid phagosome; this allowed us to follow the phagocytosis flux. After 24 hours, we found that the pHrodo emission increased over time and was maximal at 72 hours (Supplemental Figure 7D). WNT5A downregulation significantly reduced phagocytosis compared with controls (Figure 5G and Supplemental Figure 7E), and this reduction was rescued by addition of recombinant IL-6 and WNT5A proteins. Furthermore, inhibition of the CCL2 receptor, CCR2, using a specific antagonist (CCR2i) or IL-6 receptor (IL-6R) blockade using tocilizumab, a monoclonal antibody against IL-6R, effectively reduced phagocytosis in cocultures of GFP-231 with MSCs labeled with pHrodo (Figure 5G). Taken together, these data show that hybrid cell formation occurs through a phagocytosis-like mechanism mediated by a WNT5A–IL-6–CCL2 network.

### The WNT5A–IL-6–CCL2 protein-protein interaction network is associated with chemoresistance and metastasis in human TNBC.

**Figure 6:**
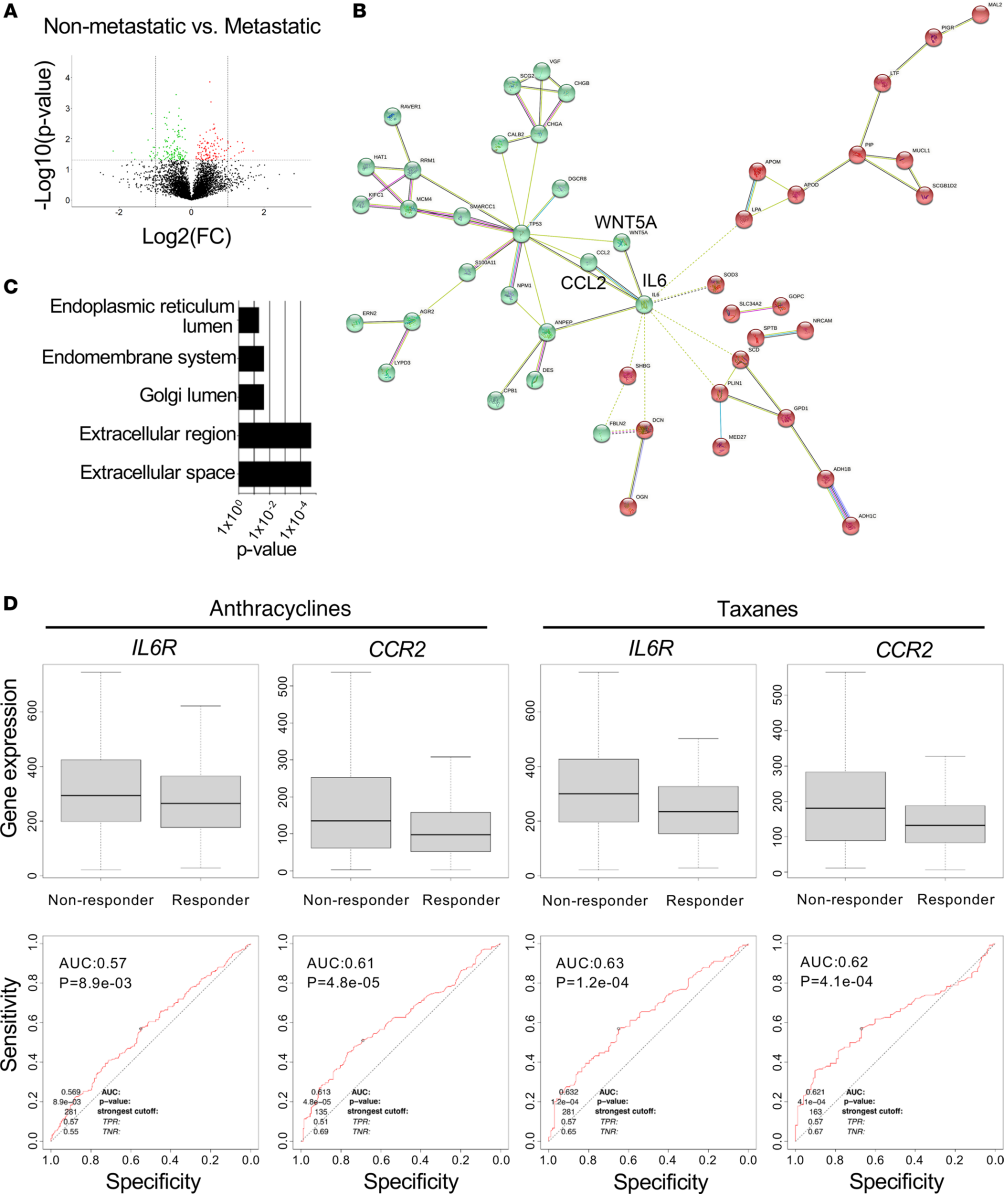
WNT5A, IL-6, and CCL2 proteins are enriched in human chemoresistant metastatic breast carcinomas. (**A**) Differential expression analysis on the proteomics profile of metastatic and nonmetastatic human TNBC tumors (*n* = 4 per group). Volcano plots show differential up- and downregulated proteins. We considered all protein in the region *P* < 0.05 and fold change > 1 and outliers within *P* < 0.001 and between –1 < fold change < 1. (**B**) Protein network graph (STRING) depicts predicted protein-protein interaction networks of WNT5A, IL-6, and CCL2 with up- and downregulated proteins in **A**. Note formation of 2 nodes (red and green), STRING analysis, PPI enrichment *P* = 6.72 × 10^–6^. (**C**) GO analysis showing the significant biological processes of the WNT5A, IL-6, and CCL2 significantly predicted protein-protein interaction network in metastatic versus nonmetastatic TNBC. (**D**) Analyses of publicly available datasets containing treatment response information on 1,329 breast cancer patients treated with antracyclines (e.g., doxorubicin) or taxanes (e.g., paclitaxel) in relationship to relapse-free survival at 5 years after treatment. Shown are box plots (top) and corresponding ROC curves. KM Plotter (Kmplot.com) was used.

To ascertain the significance of our studies to human disease, we analyzed the WNT5A–IL-6–CCL2 network in a proteomics data set that contains expression levels of more than 5,000 proteins deregulated in human TNBC that we have recently reported (16). Differential expression analyses show that chemoresistant metastatic TNBCs show upregulation of 113 proteins compared with nonmetastatic tumors (*n* = 4 independent patient samples with replicates, per group), with significant upregulation of proteins in the metabolic and nuclear processes, and with downregulation of proteins in secretory functions (Figure 6A, Supplemental Figure 8A, and Supplemental Table 3). We identified a significant WNT5A–IL-6–CCL2 predicted protein-protein interaction (PPI) network in metastatic TNBC tumors (PPI enrichment *P* = 6.72 × 10^–6^, STRING PPI analyses; Figure 6B). Gene ontology (GO) analyses of the predicted interactors in the WNT5A–IL-6–CCL2 network show significant representation of endoplasmic reticulum lumen, extracellular space, and extracellular region processes (Figure 6C). Supporting these data, independent analysis of the GSE183947 publicly available human breast cancer data set (https://www.ncbi.nlm.nih.gov/geo/query/acc.cgi?acc=GSE183947) revealed a significant positive correlation between IL-6 and CCL2 transcript levels in breast cancer metastasis compared with primary tumors (primary tumors [*r* = 0.24, *P* = 0.383] versus metastasis [*r* = 0.64, *P* = 0.0009]; Supplemental Figure 8B).

To expand on this analysis in the specific setting of breast cancer chemoresistance, we analyzed the TCGA data set using the Kaplan-Meier (KM) plotter bioinformatics platform (https://kmplot.com/analysis/). This data set contains 5-year relapse-free survival and gene expression data from patients with breast cancer treated with anthracyclines (e.g., DOXO, *n* = 383) or taxanes (e.g., PTX, *n* = 237). These analyses revealed that IL-6R and CCR2 expression is associated with response to chemotherapy. Specifically, tumors with high IL-6R or CCR2 expression had a shorter 5-year recurrence-free survival compared with tumors with low IL-6R or CCR2 (Figure 6D). Collectively, these results show that WNT5A–IL-6–CCL2 are enriched in TNBC tumors that develop metastasis and that high expression of IL-6R or CCR2 is associated with breast cancer chemoresistance and recurrence.

## Discussion

Although initially responsive to chemotherapy, TNBC patients frequently develop drug-resistant metastatic progression (3). In recent years, studies have shown that TNBC tumors are highly heterogeneous and that this property fuels chemoresistance through selection of resistant subclones (34, 35). In this study, we investigated whether the existence of breast cancer hybrid cells generated by MSC engulfment influences TNBC progression. Using clinical samples of breast cancer metastases and in vitro and in vivo models, we discovered a hybrid cell population enriched in senescent and polyploid cells that enhance TNBC chemoresistance, suggesting a potentially novel mechanism of TNBC progression.

Using a high-throughput microfluidic cell pairing chip to dynamically study the interaction between 2 single cells, we have demonstrated that BCCs can engulf MSCs, and this process results in the generation of hybrid cells with markers of BCCs and MSCs. In this study, we observed a unique hybrid population expressing pan-CK and the mesenchymal cell marker FAP in clinical tissue samples of primary invasive carcinomas and distant breast cancer metastasis that have failed chemotherapy. We show that FAP is selectively expressed by MSCs and can be used as a potential marker for the identification of BCC-engulfing MSCs in breast cancer tissues.

To evaluate the biological relevance of the hybrid cells in breast cancer drug resistance and dissemination, we generated hybrid cells in vitro culturing a panel of BCCs with MSCs, and we employed primary orthotopic and intracardiac injection models for further evaluation in vivo. We found that PTX significantly reduced primary tumor size and increased the percentage of pan-CK^+^/FAP^+^ hybrid cells. Moreover, PTX failed to reduce the number of distant metastases. In 2 independent models of breast cancer metastasis, neither PTX nor DOXO treatments reduced distant metastasis formed by hybrid cells compared with BCCs alone. Thus, we identified a hybrid cell population that enhances breast cancer chemoresistance and facilitates metastatic progression. Based on our studies, targeting this specific population could hold promise in triggering a more robust tumor response to chemotherapy and combating drug resistance.

An important finding in our study is that the hybrid cell population generated through MSC engulfment is enriched in polyploid cells with senescent properties. Although cellular senescence has been previously considered as a tumor suppressive mechanism (36), studies have shown that senescent cells maintain a metabolically active state (37) supporting tumorigenesis. Indeed, senescent cells release several mediators, including proinflammatory molecules, growth factors, and proteases that create a protumorigenic milieu. In agreement with a previous study (12), we observed that hybrid cells show increased secretion of SASP factors including IL-6, CCL2, THBS1, uPAR, and OPN. We found that approximately 50% of hybrid cells express SA–β-Gal, a well-characterized marker of senescence (23), compared with 3% of BCCs and that lung metastasis derived from hybrid cells have higher expression of SA–β-Gal than those derived from BCCs. Further supporting the senescent phenotype of hybrid cells and their relevance to metastasis, we found that the senolytic drug Navitoclax reduced hybrid cell viability and resulted in hybrid cells with reduced metastatic ability. A remaining question is whether the proliferative or the senescent cells within the hybrid cell population are responsible for their chemoresistant properties. Our data support that senescent hybrid cells remain metabolically active, secreting cytokines that allow the communication with various components of the microenvironment and potentiate the aggressiveness of breast cancer. These findings support the idea that targeting hybrid cells could serve as a promising therapeutic strategy to counteract breast cancer metastasis and overcome chemoresistance.

To elucidate the mechanism of hybrid cell formation, we investigated the involvement of WNT5A, a noncanonical signaling member of the WNT family, which we previously found to be highly expressed by BCC-engulfing MSCs (11). The role of WNT5A in tumorigenesis is not completely understood. A recent study showed that WNT5A initially supports the dissemination and seeding of melanoma cells in the lungs, activating dormancy (38). Another study demonstrated that WNT5A also induced cellular senescence in stem/progenitor cells (39). Here, we demonstrate that WNT5A downregulation reduces MSC engulfment, hybrid cell formation, and chemoresistance, supporting an important role in the maintenance of the aggressive properties of hybrid cells. Although further studies are needed to explore the role of WNT5A in hybrid cell formation in vivo, our mechanistic data demonstrate that WNT5A is a crucial player of hybrid cell formation through a phagocytosis-like mechanism. Data reported in the literature have shown that WNT5A can regulate the secretion of IL-6 and CCL2 in other types of cancer, including melanoma and non–small cell lung cancer (32, 33). Our study defines a mechanism by which WNT5A increases the production of IL-6 by BCCs, which in turn induces the secretion of CCL2 by MSCs. Using a pharmacological approach, we found that blocking IL-6R or CCR2 is sufficient to reduce hybrid cell formation. Thus, these results suggest that these proteins and their receptors may be targets to improve sensitivity to chemotherapy and/or biomarkers of therapy response. Significantly, our analysis of 620 breast cancer patients treated with anthracyclines or taxanes showed that patients with primary tumors expressing high levels of IL-6R or CCR2 are significantly less likely to respond to either chemotherapy compared with low receptor expression.

In conclusion, we provide direct evidence of a hybrid cell population in human breast cancer that can be identified by coexpression of CK and FAP proteins. Our work specifies that hybrid cells form through a phagocytosis-like mechanism mediated by a WNT5A–IL-6–CCL2 network. Our data show that the hybrid cell population is enriched in senescent cells that contribute to TNBC chemoresistance and metastatic progression. We identify predictive biomarkers and potential targets to overcome breast cancer drug resistance.

## Methods

### Tissue samples and multiplexed fluorescence IHC.

Tissue samples from primary breast cancers and breast cancer distant metastasis from 38 patients were retrieved from the Surgical Pathology files at the University of Michigan with IRB approval and reviewed by 2 pathologists. Paraffin embedded 5 μm–thick sections of each block were subjected to multiplexed fluorescence IHC using immunofluorescence. Briefly, heat-induced antigen retrieval was performed using Leica Bond Epitope Retrieval Buffer 1 (Citrate solution, pH 6.0) for 20 minutes. Nonspecific antibody binding was blocked using Novolink Protein Block (Leica, RE7280-CE) for 30 minutes. A cocktail of both primary antibodies was applied for overnight incubation at 4°C. Goat anti–mouse IgG Alexa Fluor 546 (Thermo Fisher Scientific, A11030, lot no. 2026145) and goat anti–rabbit IgG Alexa Fluor 647 (Thermo Fisher Scientific, A32733, lot no. VA294744) were applied for 60 minutes. A DAPI nuclear counterstain (blue) was applied.

Once stained, whole-slide images were generated using the Akoya Phenoimager HT, and an integrated AI-powered image quality control tool that automatically assesses focus, tissue and slide artifacts, and image quality at scale (Reveal Biosciences) was used. Positive cells in whole-slide images were identified and quantitated within stained tissue sections as the number of positive cells within the total image analysis area for each sample. The number of positive cells was counted and measured as the total amount of positive cells, total positive cell area, and percentage of positive cells within the total image analysis area for each sample. The number of pan-CK^+^ and FAP^+^ cells was recorded and served to calculate the percentage of pan-CK^+^/FAP^+^ per sample.

### Animal studies.

In vivo experiments were performed following the instructions of the NIH Guide for the Care and Use of Laboratory Animals and were allowed by the Institutional Animal Care and Use Committee at the University of Michigan. To evaluate the formation of hybrid cells in vivo, we injected GFP-MDA-MB-231 (GFP-231) single culture (1.5 × 10^6^) and GFP-MDA-MB-231 and DsRed-MSC cocultures (1:2) in the inguinal mammary fat pads of female NOD/SCID mice (*n* = 6). Two weeks after cell injections, primary orthotopic tumors were imaged in vivo, and lungs were imaged ex vivo immediately after euthanizing the mice using confocal 2-photon microscopy as described previously (40).

To investigate the effect hybrid cells on tumor growth, GFP-231 and DsRed-MSC cocultures (1:1) were orthotopically injected into the right inguinal mammary fat pad of anesthetized mice at a concentration of 1 × 10^6^ cells in matrigel (3–4 mice per group). When tumors were palpable, mice were treated with PTX (national drug codes list [NDC 63323-763-50]), 10 mg/kg i.p. every 3 days or with PBS for 15 days. In a separate experiment, GFP-231 single culture or in cocultures with equal amounts of DsRed-MSC cells were injected in the mammary fat pad, as above, at a concentration of 1.5 × 10^6^ cells (10 mice per group). Mice were treated with PTX 10 mg/kg i.p., every 3 days. The first day of PTX injection was assigned as day zero of the experiment, and mice were treated every 3 days for 3 weeks. Primary tumor size was measured twice a week using a caliper, and mice were euthanized (tumor volume = (length × width^2^)/2). Metastases were quantified by GFP pixels in the lungs using ImageJ (NIH) and confirmed by histology.

To investigate distant dissemination in the absence of primary tumor, Luc–GFP-231 were cultured alone or with DsRed-MSCs for 72 hours and injected intracardially in 8-week-old female severe combined immunodeficient mice (NOD Cg-Prkdc ll2rgSzJ [NSG], stock no. 005557, The Jackson Laboratory) at a concentration of 1 × 10^5^cells (GFP-231 alone) or 1.5 × 10^5^ (GFP-231/DsRed-MSCs) resuspended in 50 μL of PBS (*n* = 10 mice per group). At day 20, mice were divided into 2 groups (*n* = 3–4 mice per group) and treated every 3 days i.p with DOXO dissolved in saline at doses of 4 mg/kg or vehicle. Metastases were monitored using bioluminescence imaging as previously described (11). Bioluminescence images were acquired using the IVIS imaging system (Xenogen) within approximately 2–5 minutes after injection. Analysis was performed as described (13). Mice were sacrificed and necropsied at day 27. Metastases were identified by GFP fluorescence microscopy immediately after collecting the tissues at necropsy. The number of metastases per mice per group was quantified using ImageJ. To study distant dissemination in a immunocompetent mouse model, Luc–GFP-CCN6–KO were cultured alone or with DsRed-MSCs for 5 days and treated with vehicle or Navitoclax 5 μM for 48 hours prior to being injected intracardially in 8-week-old FVB mice at the concentration of 1 × 10^5^cells (Luc–GFP-CCN6–KO) (*n* = 6–14 mice per group). After 2 days, mice injected with untreated Luc–GFP-CCN6–KO in single culture or coculture were treated with vehicle or PTX 10 mg/kg for 10 days. Mice were sacrificed and necropsied at day 13.

### Reagents and antibodies.

FAP antibody was purchased from Cell Signaling (catalog 66562). DOXO (catalog D1515), PTX (catalog T7402), and CCR2 antagonist (catalog 227016) were purchased from Sigma-Aldrich. Navitoclax (ABT-263) (catalog S1001) was purchased from Selleckchem. Recombinant IL-6 protein (catalog 7270-IL), recombinant WNT5A protein were acquired from R&D System (catalog 645-WN). Tocilizumab was acquired from Novus Biologicals (catalog NBP2-75193). Puromycin was acquired from Thermo Fisher Scientific (catalog A11138). Cell Signaling Technology antibodies contributed: anti–PKC-α (catalog 59754S), anti–p-PKC-α/β II (catalog 9375S), anti–p-PLC-γ1 (catalog 8713S), anti–PLC-γ1 XP (catalog 5690S), anti–p-RAC1/cdc42 (Ser71) (catalog 2461), anti-RAC1/2/3 (catalog 2465), anti-WNT5A/B (catalog MA5-15502), and anti–cleaved caspase-3 (catalog 9661S). Mouse monoclonal β-actin–HRP (catalog 47778, Santa Cruz Biotechnology) was used as loading control. Anti-Ki67 was acquired by BD Horizon (catalog 565929).

### Cell culture.

BCC lines MDA-MB-231, MDA-MB–436, MCF-7, and MDA-MB-468 were purchased from the American Type Culture Collection and grown under recommended conditions. Patient-derived cancer (T4) as well as MSCs isolated from fresh human breast cancer metastasis to a supraclavicular lymph node (LN-MSCs) and to the liver (Lv-MSCs) were isolated and characterized in our lab (13). CCN6-KO cells derived from MMTV-Cre;Ccn6^fl/fl^ mammary breast carcinomas developed and validated in our lab (15) were cultured with DsRed-MSCs for 7 days. Cell lines were authenticated using STR profiling and were tested for mycoplasma infection using Sigma LookOut Mycoplasma PCR Detection Kit (catalog MP0035). BCCs and MSCs were labeled with GFP and DsRed, respectively. In another set of experiments, BCCs were labeled with DsRed. To generate hybrid cells, 2 × 10^5^ DsRed-MSCs and 1 × 10^5^ GFP -BCCs were cultured in complete medium (50% of BCC medium and 50% of MSC medium) for 72 hours or 7 days as indicated.

Human WNT5A knockdown was achieved using shRNA (MISSION shRNA, Sigma-Aldrich; Broad Institute constructs TRCN0000288987 and TRCN0000296083 for WNT5A) as previously reported (41). Background control was Lenti-PuroEMPTY-VSVG. Cells were selected with 1 μg/mL puromycin over time to eliminate uninfected cells. WNT5A protein expression in stable clones were evaluated by immunoblotting.

### Fluorescence imaging.

DsRed-MSCs and GFP-MDA-MB-231, GFP-MDA-MB-436, GFP-T4, or GFP-CCN6–KO BCCs were seeded in a 6-multiwell plate and cultured in complete medium for 72 hours. In another set of experiments, BCCs were cultured alone or with DsRed-MSCs in 24-multiwell plate for 48 hours and then treated with DOXO or PTX as indicated. GFP and DsRed pixels were quantified by viewing 5 separate fields at 10× magnification (OLYMPUS IX-71), using ImageJ software. GFP and DsRed signals from DsRed-MSCs and 1 × 10^5^ GFP-MDA-MB-231 coculture were also captured using an inverted spinning disk confocal system head from Yokogawa (Yokogawa CSU-X1 Spinning Disk Unit, Yokogawa Electric Corporation), a cooled digital CCD camera (AxioCamMRm, Zeiss), and 2 laser lines (488 nm and 561 nm, Zeiss) attached to the spinning disc confocal scan head. The system is enclosed in a custom environmental chamber for temperature (37°C) and CO_2_ (5%) control. Images were recorded using a Plan-Apochromat ×63/1.44 DIC oil objective operated via the Zen 2012 (Blue edition) software platform.

### ELISA.

Levels of IL-6, CCL2, OPN, THBS-1, and uPAR were measured in supernatants derived from BCCs (MDA-MB-231, MDA-MB-436, T4) and MSCs cultured alone or in coculture for 72 hours. Supernatants from MSCs and BCCs plated in single culture were diluted 1:1 and used as control. Levels of CCL2 were measured in supernatants derived from MSCs after treatment with IL-6 recombinant protein (rh) 30 ng/mL for 24 hours. All ELISAs were performed using human ELISA kits according to manufacturer’s instructions (R&D Systems).

### Cell senescence studies.

MDA-MB-231 TNBC cells and patient-derived MSCs cells isolated and validated were used to evaluate cellular senescence. Single and coculture 1:1 ratio of MDA-MB-231 and MSCs were seeded in 6-well plates and cultured for 1 week, before being subjected to a β-Gal Staining Kit (Cell Signaling Technology, 9860). Following manufacturer’s instructions, cells were fixed for 15 minutes in the fixative solution at room temperature. Cells were washed twice with PBS and incubated with β-Gal staining solution at 37°C overnight in a dry incubator (no CO_2_). For each sample of the single cultures and cocultures, 10 randomly chosen fields were photographed using a camera-equipped bright field microscope (Olympus) at 10× of magnification. Data were plotted as the percentage of cells with β-Gal activity. Cells were also subjected to a Quantitative Cellular Senescence Assay Kit (SA–β-Gal, Fluorometric) (Cell Biolabs Inc., CBA-232). Flow cytometry and epifluorescence were used for β-Gal activity detection. MDA-MB-231 cells labeled with GFP or with DsRed and unlabeled MSCs were plated as above and pretreated for 2 hours at 37°C with the 1× solution. SA–β-Gal substrate solution was added to the cells and incubated overnight at 37°C. For flow cytometry cells were collected and washed in cold PBS, spun-down at low speedy for 5 minutes, and resuspended the pellets in PBS + DAPI with 2% of FBS. For epifluorescence analysis, the cells were fixed in 4% paraformaldehyde for 30 minutes at room temperature, washed 3 time with PBS, and counterstained with DAPI (60× of magnification). All experiments were performed at least 3 times with similar results. Data were analyzed by 2-tailed unpaired Student’s t test for the SA–β-Gal activity assay and by 1-way ANOVA with Tukey’s multiple-comparison test for the SA–β-Gal flow cytometry assay. IHC for β-Gal staining was performed using citrate retrieval at pH 6.0 and a pressure cooker. Anti–GLB1/β-Gal antibody (ab203749, Abcam) was used at 1:250 for 1 hour. We used HRP Polymer for 30 minutes and hematoxylin for 5 minutes. Quantification of immunostaining was evaluated by histopathology and quantified in 5 different fields per condition using ImageJ.

### Human cytokine array.

Human XL Cytokine Array Kits (R&D Systems) were used to analyze the secreted proteins in the supernatants derived from MSCs and MDA-MB-231 in single culture or direct or transwell coculture as described in the manufacturer’s protocol. Transwell cocultures were established using transwell inserts (0.4 μm pore polycarbonate membrane insert Corning, 3412), loading MSCs in the upper inserts and MDA-MB-231 into the lower compartment of the culture well. The intensity of selected spots was quantified using ImageJ software.

### Immunoblot analysis.

Immunoblot analyses were carried out with 100 μg of whole-cell extract derived as previously reported (41). Membranes were blocked and incubated with primary antibodies in 4% milk (Sigma-Aldrich, A3059) in TBS-T (Bio-Rad, 161–0372, with 0.05% Tween20) at 4°C overnight.

### Flow cytometry analysis.

The percentage of GFP^+^ and GFP^+^/DsRed^+^ population in DsRed-MSCs cultured with BCCs for 72 hours was determined by FACS analyses. In another set of experiments, MSCs cultured with BCCs for 48 hours were treated with DOXO or PTX as described. Single-cell pictures were taken from the DsRed^+^/GFP^+^ population using Life Imaging Stream Flow Cytometry. All flow cytometry analyses were completed using the UM Flow Cytometry Core (Ann Arbor, Michigan, USA), in triplicate.

### Cell cycle analysis using flow cytometer.

Cells were collected by trypsinization, resuspended in ice-cold PBS, and fixed by adding ice-cold ethanol. After 20 minutes of incubation, cells were centrifuged for 10 minutes at 4°C and 244*g*, resuspended in 0.5 mL PBS/RNase solution containing 50 μg/mL DAPI for 20 minutes in the dark and analyzed by FACS. To determine the percentage of Ki67^lo^ cells in G0-G1 phase of the cell cycle, cells were fixed in ethanol and stained in 100 μL of Brilliant Stain Buffer (BD Horizon) with anti-Ki67 for 30 minutes in the dark. After 2 washes in the Brilliant Stain Buffer, cells were resuspended in regular medium, stained with Vybrant DyeCycle Ruby (Invitrogen), and analyzed by FACS by Bio-Rad ZE5 #2 Cell Analyzer (Bio-Rad) at the UM Flow Cytometry Core.

### Microfluidic chip design and fabrication.

The high-throughput cellular engulfing device was built by a polydimethylsiloxane (PDMS) piece with microfluidic patterns bonded to a glass slide. The PDMS was patterned by standard soft lithography. The SU-8 mold used for soft-lithography was created by a 3-layer photolithography process with 10 μm–, 40 μm–, and 100 μm–thick SU-8 (Microchem) following the manufacturer’s protocol. The pattern was designed using computer aided design software (AutoCAD 2015, Autodesk), and the masks were made by a mask-making instrument (μPG 101, Heidelberg Instruments). The SU-8 mold was treated by vaporized trichloro(3,3,3-trifluoropropyl)silane (452807, Sigma-Aldrich) under vacuum overnight to promote the release of cured PDMS. PDMS (Sylgard 184, Dow Corning) was prepared by mixing with 10:1 elastomer/curing agent (w/w) ratio, poured on SU-8 molds, and cured at 80°C for 4 hours before peeling. Inlet and outlet holes were created by biopsy punch cutting. The PDMS piece with microfluidic channel structures and glass slide were treated using oxygen plasma (100W for 60 seconds) and bonded. The devices were heated at 80°C overnight to ensure bonding quality.

### Cell engulfment on chip.

We employed the microfluidic cell pairing device as reported in our previous study (11). Briefly, the microfluidic chip was sanitized by UV radiation prior to use to ensure aseptic conditions. Before cell loading, collagen solution (1.45 mL Collagen [Collagen Type 1, 354236, BD Biosciences], 0.1 mL acetic acid in 50 mL DI Water) was flowed through the device for 1 hour to coat collagen on the substrate to enhance cell adhesion. Devices were then rinsed with PBS for 1 hour to remove the residual collagen solution. Cancer cells and MSCs were harvested from culture plates and resuspended in culture media to a concentration of 2 × 10^4^ cells/mL. Cancer cell suspension and MSC suspension were mixed 1:1 by volume. In total, 100 μL of the cell suspension solution was then pipetted into the chip inlet, and the cells were driven into the chip by gravity flow. After the cell loading for 5 minutes, the cell suspension solution was replaced by 100 μL of cell culture media to stop cell loading, and the chip was imaged to readout cell-pairing scenarios initially. The entire chip was placed into a cell culture incubator. Cells were treated with control IgG or anti-WNT5A/B, or they were untreated. After 72 hours, cells were treated with vehicle or PTX 10 μM for 48 hours. Ten cells for each condition were counted, in biological triplicate. Engulfment rate was measured based on the final status of each device.

### Phagocytosis assay.

Phagocytosis assay was performed using Incucyte pHrodo Red Cell Labeling Kit (Essen BioScience, 4649), according to the manufacturer’s instruction. Briefly, 1 × 10^5^ GFP-MDA-MB-231 or -T4 were seeded in 24-well plate for 24 hours and incubated with 2 × 10^5^ MSCs labeled with pHrodo dye according to the manufacturer’s instruction. In a set of experiments, GFP-MDA-MB-231 were treated, as described for 30 minutes before MSCs were plated on top of BCCs. Fluorescence was photographed with OLYMPUS IX-71 microscope at 20***×*** magnification. Integrated red intensity above threshold standardized of pHrodo MSCs was obtained using ImageJ software and normalized to number of cells.

### Whole-transcriptome sequencing.

We retrieved 100,000–200,000 MDA-MB-231 and MSCs for next-generation whole-transcriptome sequencing to identify differentially expressed genes. The RNA was extracted as previously described (11). We identified genes with significant differences in expression between MSCs and BCCs as defined by *P* < 0.01 and logarithmic fold change > 0.5. For visualization of gene expression signature, R-based Seurat (42) was used in analysis. For pathway analysis, top-ranked significantly differential genes were applied to Enrichr (http://amp.pharm.mssm.edu/Enrichr/), and the pathway data set of NCI-Nature 2016 was used. The data have been deposited on GEO (accession no. GSE92485)

### Statistics.

Data are represented as mean ± SEM, and experiments were performed at least in triplicate. The number of replicates is indicated in the dot plots in the figures and corresponds to biological replicates. Differences between 2 groups were examined by 2-tailed unpaired Student’s *t* test. One-way ANOVA or 2-way ANOVA with Tukey’s or Dunn’s multiple comparison test were used to compare more than 2 groups, as indicated. *P* value of less than 0.05 was considered significant.

### Study approval.

This study was conducted with approval from the University of Michigan UCUCA protocol no. PRO00010731 and IRB protocol no. HUM00050330.

### Data availability.

Values for all data points in graphs are reported in the Supporting Data Values file.

## Author contributions

MEG, GA, and CGK formulated the hypothesis and study design. MEG, GA, AP, BB, AE, and CGK performed experiments and analyzed and interpreted data. GDL, JMB, and MEG designed and performed the in vivo confocal microscopy studies. YCC, YC, and EY developed and performed the microfluidic engulfing assay. SD and AIN performed and analyzed the TMT proteomics. SKK and SV analyzed publicly available human breast cancer data sets. LM, DB, and SA contributed to aspects of the study design. GA, MEG, and CGK wrote the paper.

## Supplementary Material

Supplemental data

Supplemental video 1

Supporting data values

## Version 1 08/22/2023

In-Press Preview

## Version 2 09/22/2023

Electronic publication
