# Supplementary material for: A hybrid breast cancer/mesenchymal stem cell population enhances chemoresistance and metastasis
Source: JCI Insight. 2023 Sep 22;8(18):e164216. doi: 10.1172/jci.insight.164216 (PMC10561721; doi:10.1172/jci.insight.164216)
Supplement: Supplemental data [file jciinsight-8-164216-s147.pdf]

Supplementary Figure 1

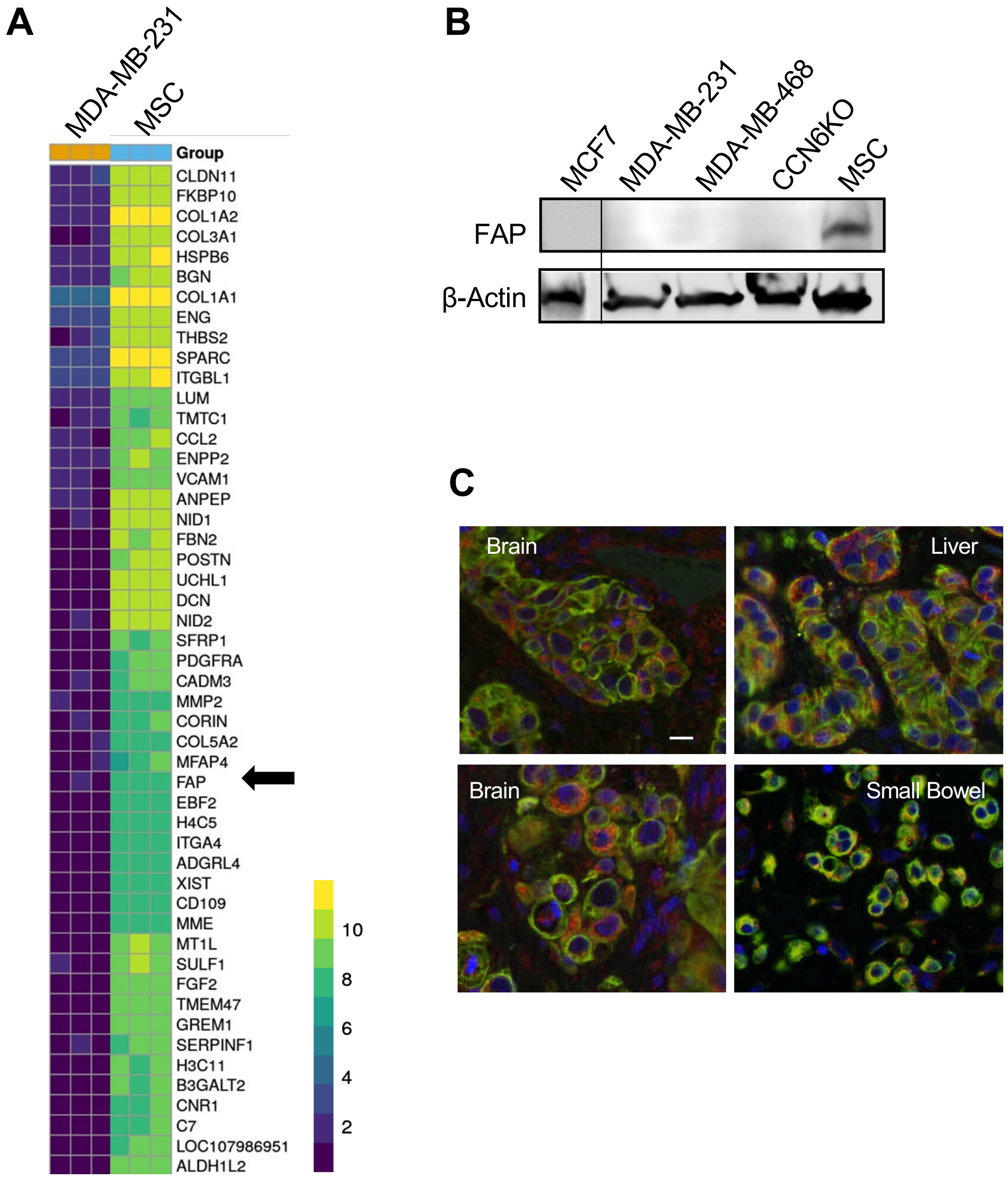

Supplementary Figure 1. MSCs derived from human breast cancer metastasis samples express FAP.

a. Heat map of RNA sequencing showing top 50 genes upregulated in MSCs compared to MDA-MB-231 breast cancer cells.

b. Western blot for fibroblast activating protein-α (FAP) in MSCs and breast cancer cells. β-actin was used as control for equal loading and transfer.

c. Representative IF images of samples of human breast cancer metastasis showing the presence of hybrid cancer cells co-expressing panCK and FAP. Scale bar, 30 um

Supplementary Figure 2

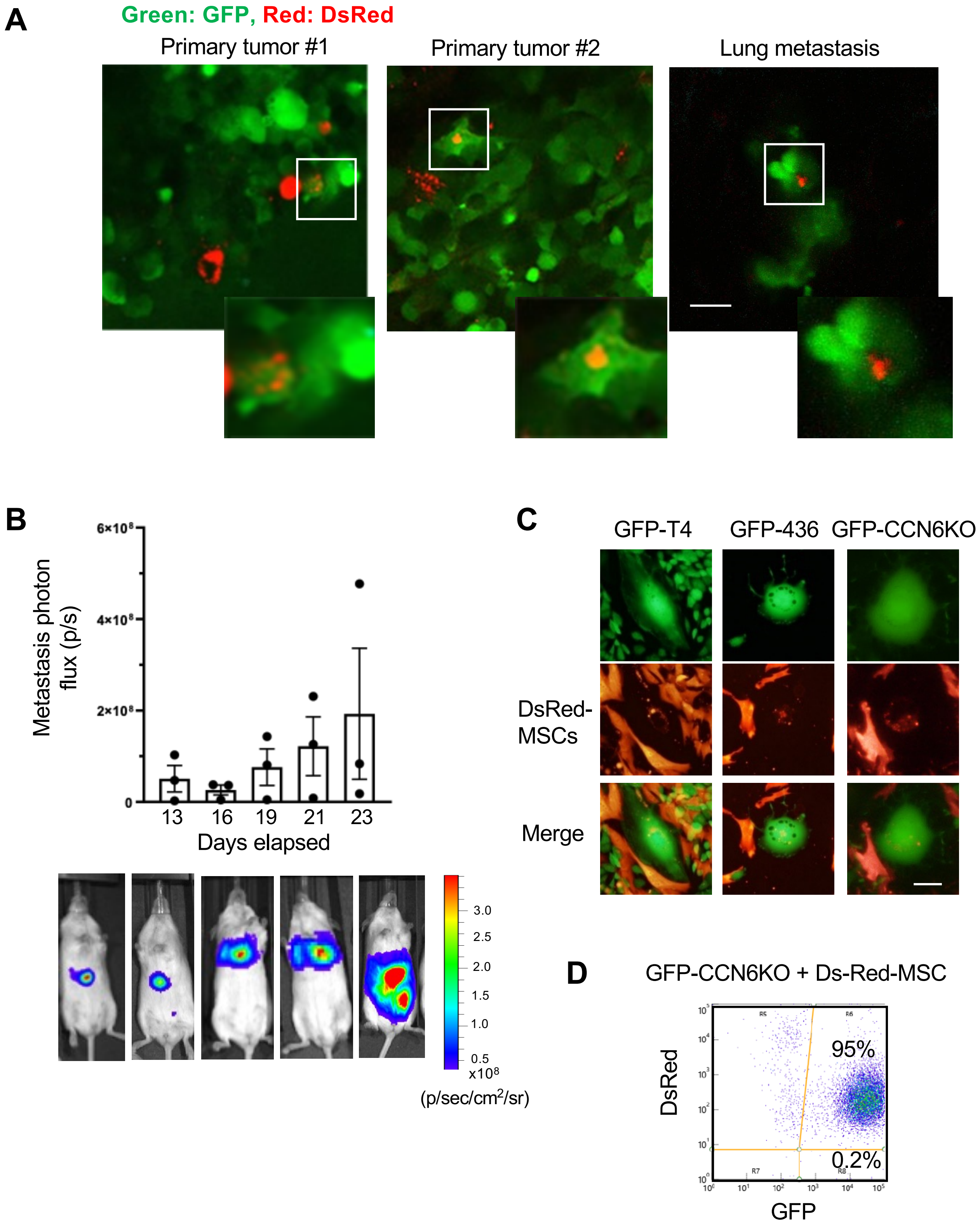

Supplementary Figure 2. Identification of Hybrid GFP+/DsRed+ cells in xenografts and lung metastasis *in vivo* and in coculture.

A. Visualization of engulfment in orthotopic xenografts and lung metastases. GFP-231 and DsRed-MSC were injected in the mammary fat pads of NOD/SCID mice. Two photon microscopy fluorescent images with Z stack were obtained after 2 weeks of cell injection.

B. GFP-MDA-MB-231 labeled with Luciferase were cultured with DsRed-MSCs for 3 days. GFP+/DsRed+ cells were sorted by flow cytometry and intracardially injected in NOD/SCID mice (n=3 mice per group). Top, Quantification of bioluminescence imaging of metastatic burden at the indicated time points. *Bottom*, representative images.

C. Fluorescence microscopy of hybrid cells in co-cultures of DsRed-MSCs and GFP-T4,-MDA-MB-436,-CCN6KO breast cancer cells. Scale bars, 30μm.

D. Flow cytometry analysis of GFP-CCN6KO cells cultured with DsRed-MSCs for 7 days. Shown is the quantification of GFP+/DsRed+ and GFP+ cells.

Supplementary Figure 3

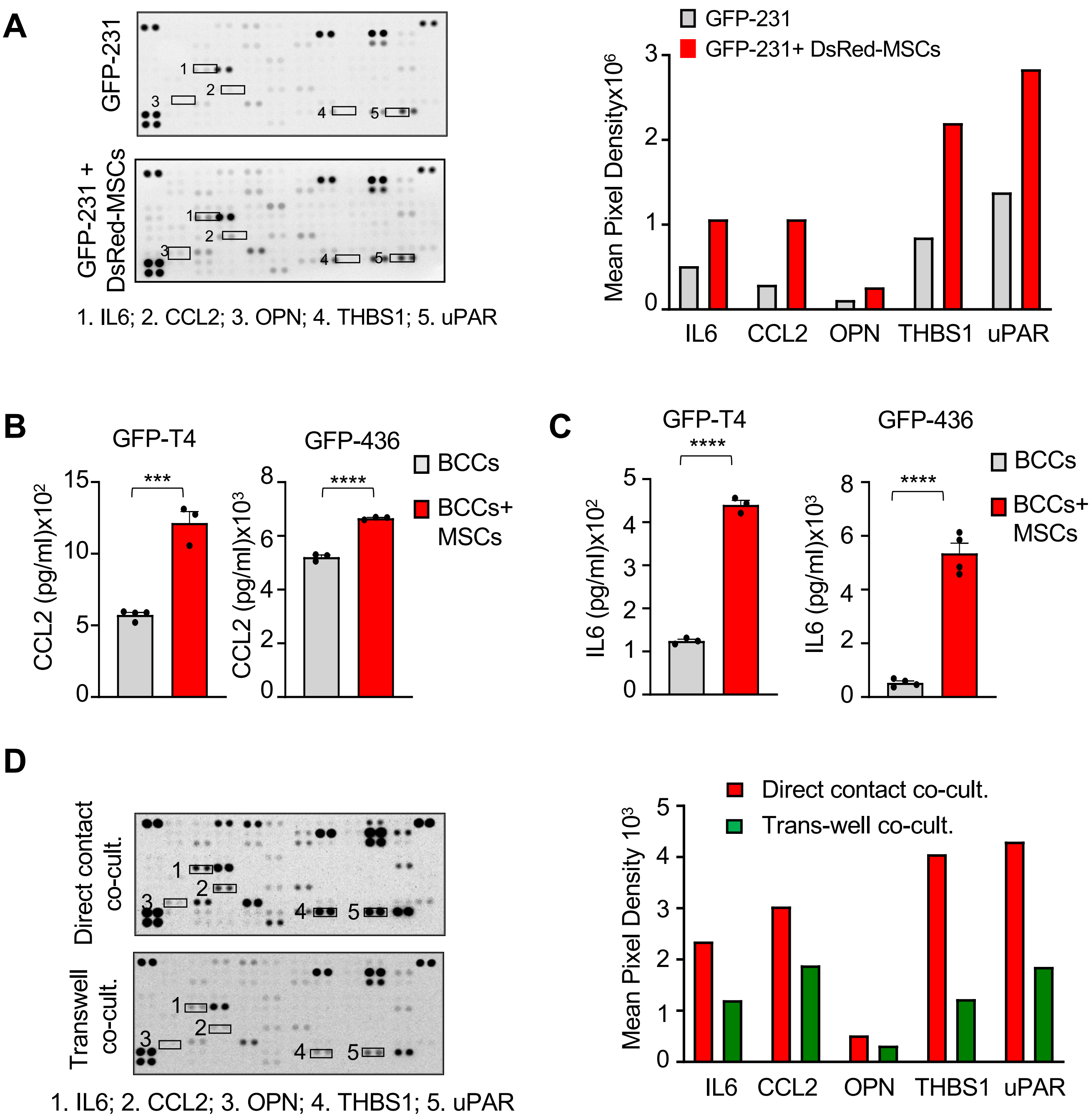

Supplementary Figure 3. Hybrid cells exhibit a senescence associated secretome.

A. *Left,* Human cytokine arrays in supernatants of GFP-231 or co-cultures of GFP-231 with DsRed-MSCs (1:1). *Right,* Raw numerical densitometry data were extracted, and the background subtracted using ImageJ. Results were shown as mean pixel density.

B-C. ELISA analyses showing the quantification of CCL2 (**B**) and IL6 (**C**) in supernatants of GFP-T4 or GFP–MDA-MB-436 (GFP-436) BCCs alone or in co-cultures with MSCs (1:1). Data are expressed as individual values with mean ± SEM analyzed with unpaired Student’s t test

D. Human cytokine array in the supernatants of GFP-231 and DsRed-MSCs cultured in direct contact co-culture or using a trans-well system. Raw numerical densitometry data were extracted, and the background subtracted. Results are shown as mean pixel density. *p<0.05, ***p<0.0005; ****p<0.0001.

Supplementary Figure 4

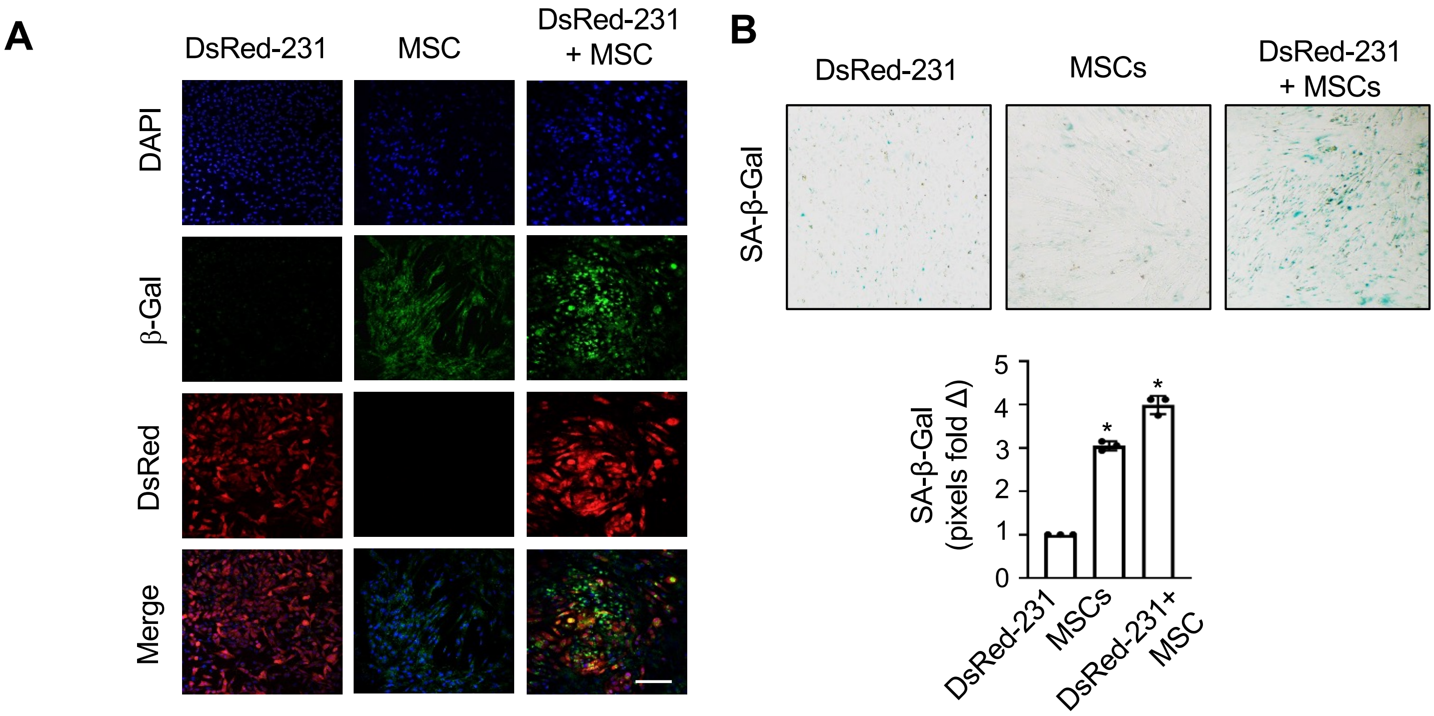

Supplementary Figure 4. Hybrid cells have features of senescent cells.

A. Immunofluorescence of senescence-associated β-galactosidase (green) in single cultures and co-cultures of DsRed-MDA-MB-231 (DsRed-231) and MSCs. Scale bar, 200mm. 400x magnification.

B. Senescence-associated (SA)-β-galactosidase assay in single cultures and co-cultures of DsRed-231 and MSCs. 200x magnification. Bar graph shows the quantification of the SA-β-Gal in five different fields per condition using ImageJ.

Data are expressed as individual values with mean ± SEM analyzed with unpaired Student's t test. **p*<0.05, ****p*<0.0005.

Supplementary Figure 5

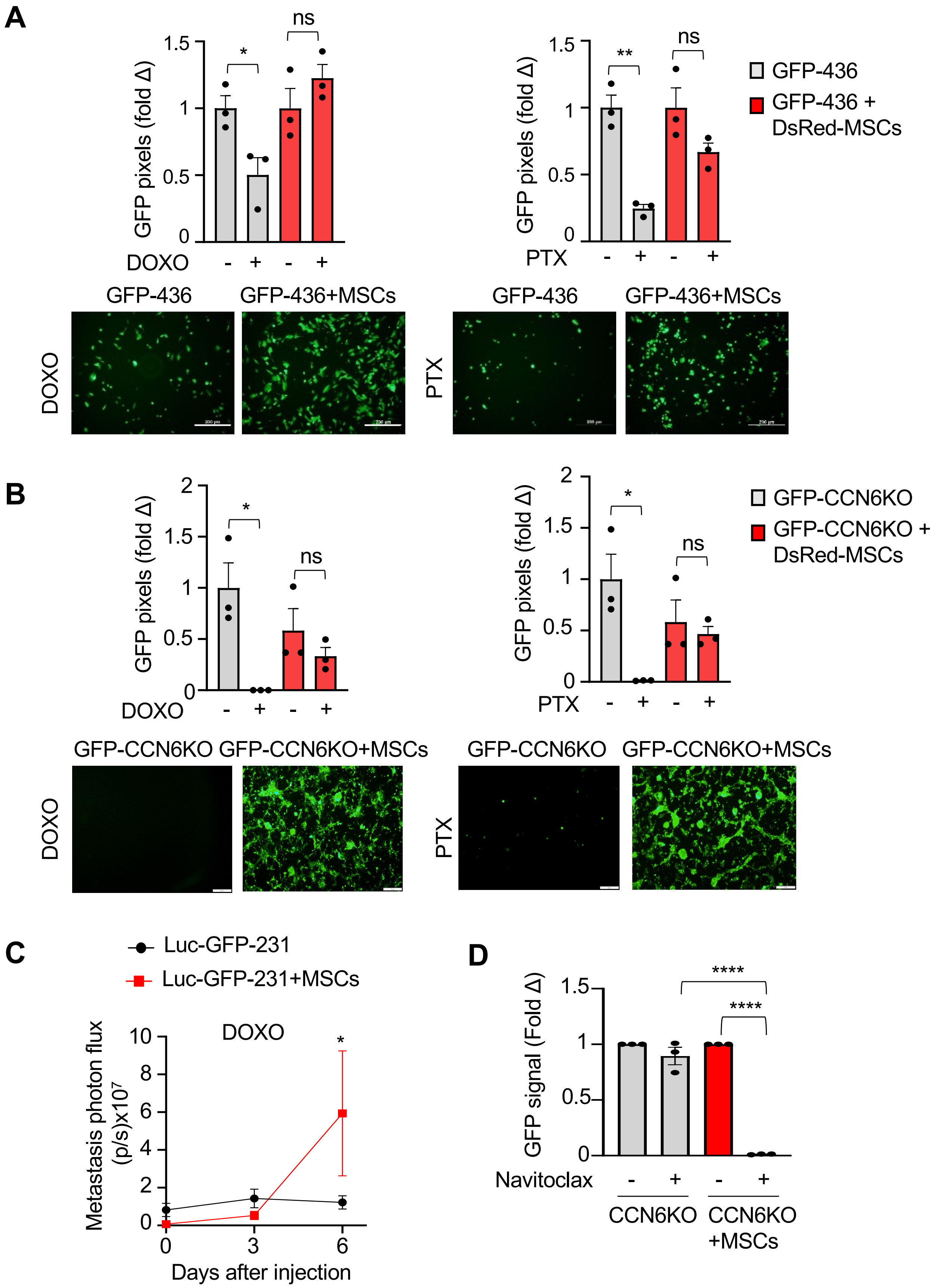

Supplementary Figure 5. Hybrid cells are resistant to drug treatment.

A. GFP signal of GFP-436 cells cultured alone or co-cultured with DsRed-MSCs for 48 h and treated with vehicle (-), doxorubicin (DOXO) 1 μM. or paclitaxel (PTX) 10 μM for 24 h. GFP was quantified in five different fields per condition using ImageJ and shown as fold change to vehicle treated cells. Representative pictures of GFP signal upon drug treatment.

B. GFP signal of GFP-CCN6KO cells cultured alone or co-cultured with DsRed-MSCs for 7 days , treated with vehicle (-), doxorubicin (DOXO) 1 μM. or paclitaxel (PTX) 10 μM for 24 h. GFP was quantified in five different fields per condition using ImageJ and shown as fold change to vehicle treated cells. Representative pictures of GFP signal upon drug treatment.

C. Metastatic burden in mice injected with Luc-GFP-231 alone cultured with MSCs after DOXO treatment 4 mg/kg (n=5 mice per group) every three days.

D. GFP signal of GFP-CCN6KO cells cultured alone or with DsRed-MSCs for 5 days, treated with Navitoclax 5 μM or vehicle for 48 h. GFP was quantified in five different fields per condition using ImageJ and shown as fold change to vehicle treated cells.

In A and B one-way Anova with Turkey's multiple comparison test was employed; in C 2-tailed unpaired Student's t test was employed; in D two-way Anova with Turkey's multiple comparison test was employed. Data are expressed as individual values with mean ± SEM **p*<0.05; *****p*<0.0001; ns: not significant.

Supplementary Figure 6

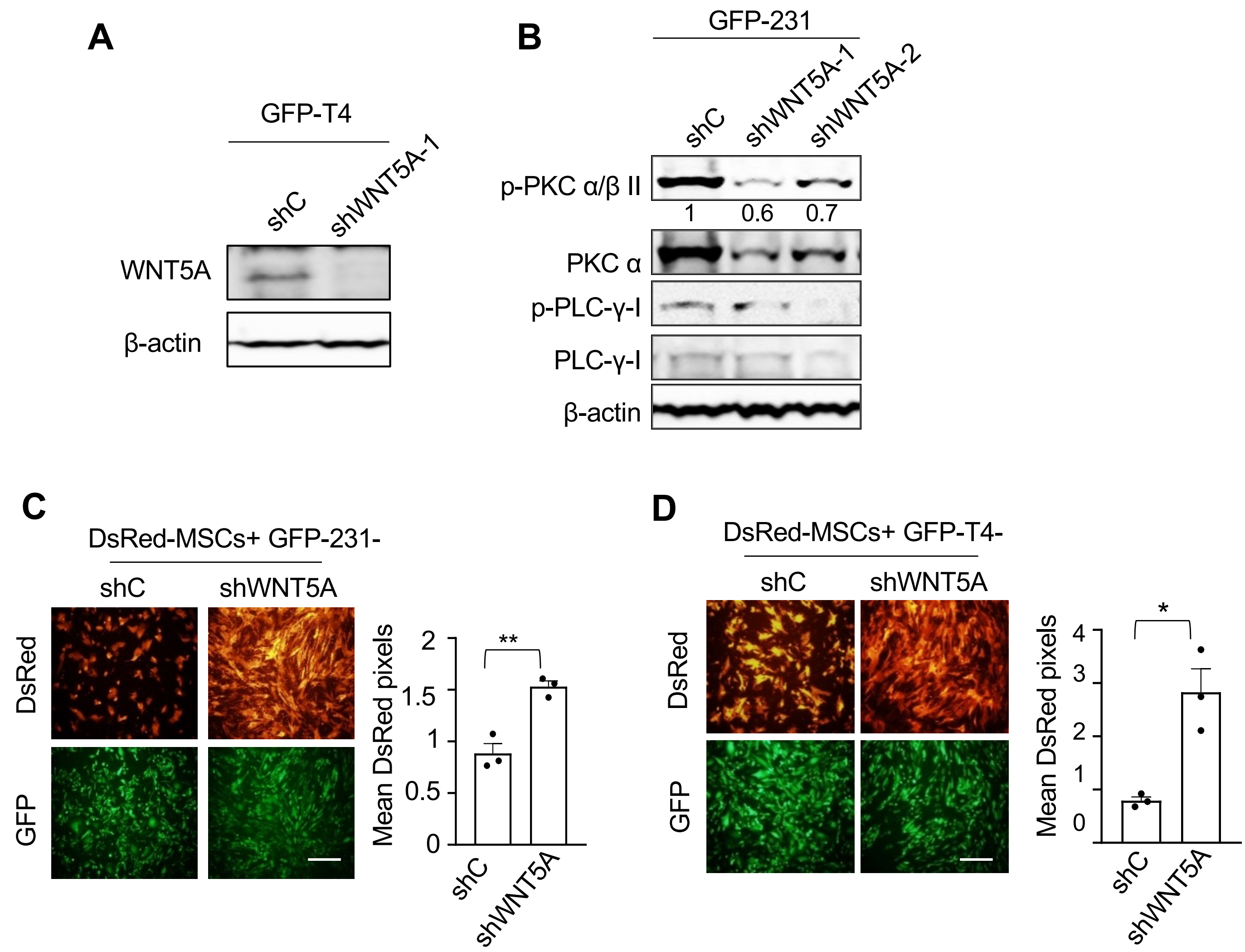

Supplementary Figure 6. **WNT5A downregulation in BCCs reduces MSC engulfment.**
A. Immunoblot of WNT5A in GFP-T4 BCCs stably transduced with shWNT5A-sh#1 or lentivirus control (-shC).
B. Immunoblot of indicated proteins in GFP-231-shWNT5A (#1 and #2) or lentivirus control (231-GFP-shC). β-Actin was used as a control for equal loading and transfer for **A** and **B**. Numbers below the blots represent the average fold change over GFP-231-shC.
C-D. Immunofluorescence microscopy of GFP-231- (**D**) and GFP-T4- (**F**) cells with shRNA knockdown of WNT5A or shC, cultured with DsRed-MSCs for 72 h. Scale bars, 200mm. Bar graph shows the quantification of DsRed signal normalized to GFP signal. DsRed and GFP signals were quantified in three different field per condition using ImageJ.
Data are expressed as individual values with mean ± SEM analyzed with 2-tailed unpaired Student's t test was employed. *p<0.05, **p<0.005.

Supplementary Figure 7

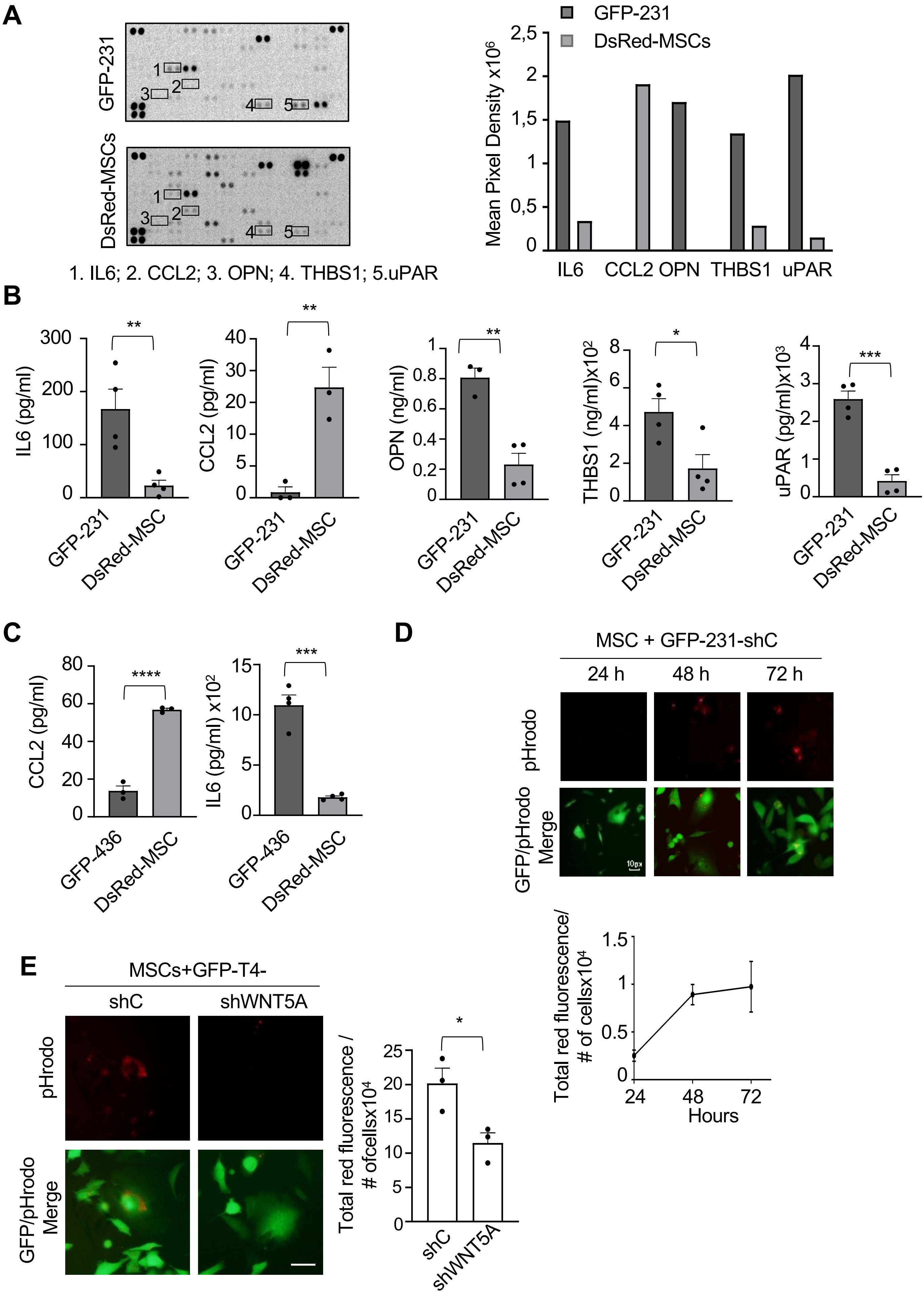

Supplementary Figure 7. Differential SASP protein secretion in BCCs and MSCs.

A. Human cytokine arrays in supernatants of GFP-231 and DsRed-MSCs. Raw numerical densitometry data were extracted, and the background subtracted. Results are shown as mean pixel density.

B-C. ELISA analyses for indicated proteins in the supernatant of DsRed-MSCs, GFP-231 (**B**), and GFP-436 (**C**).

D. Representative pHrodo and merge fluorescence images captured over a time course of GFP-231 engulfing pHrodo- labeled MSCs. Graph shows the quantification of the total red fluorescence from three different fields using ImageJ, normalized to the cell number. Scale bar, 20μm.

E. Representative pHrodo and merge fluorescence images of GFP-T4-shC, -shWNT5A engulfing pHrodo labeled MSCs after 48h. Bar graph shows the quantification of total red fluorescence normalized to the cell number. pHrodo signal was quantified in tree different field per condition using ImageJ. Scale bars, 30μm. Data are expressed as individual values with mean ± SEM analyzed with 2-tailed unpaired Student's t test was employed. *p<0.05, **p<0.005; ***p<0.0005, ****p<0.0001.

Supplementary Figure 8

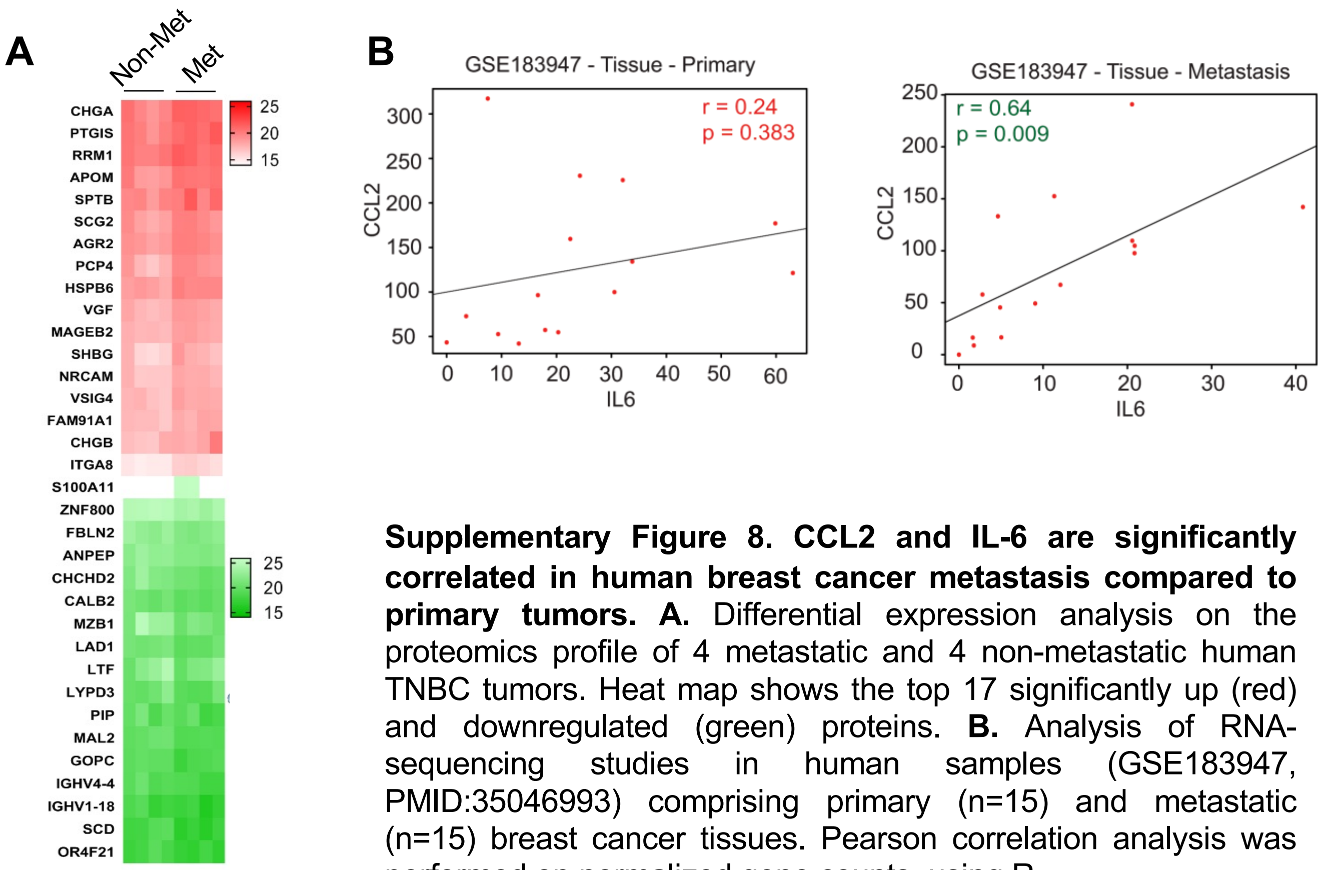

Supplementary Figure 8. CCL2 and IL-6 are significantly correlated in human breast cancer metastasis compared to primary tumors. A. Differential expression analysis on the proteomics profile of 4 metastatic and 4 non-metastatic human TNBC tumors. Heat map shows the top 17 significantly up (red) and downregulated (green) proteins. **B.** Analysis of RNA-sequencing studies in human samples (GSE183947, PMID:35046993) comprising primary (n=15) and metastatic (n=15) breast cancer tissues. Pearson correlation analysis was performed on normalized gene counts, using R.

Supplementary Table 1. Table summarizes the features of the primary breast carcinomas and percentage of CK+/FAP+ cells as well as total cells analyzed per sample.

Case	Histological type	Tumor grade	Tumor size (cm)	Positive LN / total LN (n)	ER	HER2	CK+/FAP+ cells (%)	Total # of CK+ cells
1	Ductal	2	1.7	2/5	+	-	12.3	80401
2	Lobular	2	4.3	7/22	+	-	8.37	43674
3	Lobular	1			+	-	24.51	19089
4	Ductal				+	+	2.4	18000
5	Ductal	3	0.9	38/38	+	-	16.32	28749
6	Ductal	3	3.2	9/23	-	-	0.71	14227
7	Ductal	1			+	-	7.91	1254
8	Ductal	3	2.6	2/21	+	-	1.7	16947
9	Ductal	3	4.6		+	-	11.53	241678
10	Ductal	4	2		+	-	2.49	12951
11	Metaplastic	3	1.8		-	-	0.6	134368
12	Metaplastic	3	5		-	-	0.14	425120
13	Metaplastic	3	2		-	-	1.92	7003
14	Metaplastic	3	2.3		-	-	1.12	24748
15	Ductal	3	1.8	15/22			12.7	12579
16	Ductal	2	2.3	0/3	+	-	14.84	201736
17	Ductal			25/29	+	-	0.1	6844
18	Lobular	2	1.1	1/10	+	-	7.93	12871
19	Ductal	2	1.9	1/10	+	-	4.97	242684
20	Ductal	2	5	5/26			5.96	139350

Supplementary Table 2. Table summarizes the features of the metastatic breast carcinomas and percentage of CK+/FAP+ cells as well as total cells analyzed per sample.

Case	Metastasis site	Histological type	ER	HER2	CK+/FAP+ cells (%)	Total # of CK+ cells
1	Brain	Metaplastic	-	-	32	81556
2	Brain	Ductal	+	+	42	231784
3	Brain	Ductal	-	-	39	80056
4	Brain	Ductal	-	-	31	142371
5	Brain	Ductal	-	-	42	169757
6	Brain	Ductal & lobular	-	+	13	381235
7	Brain	Ductal	-	-	61	60236
8	Liver	Ductal	-	+	32	267326
9	Intestine	Lobular	+	-	36	80873
10	Bone	Ductal	+	-	1.79	24390
11	Bone	Lobular	+	-	0.04	1332
12	Bone	Lobular	+	-	0.99	11465
13	Bone	Ductal	+	+	15.56	6313
14	Bone	Ductal	+	-	10.17	9413
15	Bone	Ductal	-	-	2.23	1626
16	Bone	Ductal	+	-	0.53	83
17	Bone	Ductal	+	+	7.7	461907
18	Bone	Ductal	+	-	7	8124

Supplementary Table 3. Table shows the gene ontology (GO) enrichment analysis for the top biological processes by fold enrichment, for up and downregulated processes in metastatic vs. non-metastatic human TNBCs

	GO process	p-value
Upregulated	Cellular nitrogen metabolic process	0.0125
	Metabolic process	0.0159
	DNA replication	0.0159
	Nuclear metabolism	0.0159
	Cellular component biogenesis	0.0321
	DNA metabolic process	0.0424
Downregulated	Vesicle-mediated transport	3.35E-06
	Endoplasmic reticulum stress	3.16E-05
	ATP biosynthetic process	5.53E-05
	ATP metabolic process	0.0015
	Golgi vesicle transport	0.002
